# Identification and Quantification of Volatile Ramson-Derived Metabolites in Humans

**DOI:** 10.3389/fchem.2018.00410

**Published:** 2018-09-11

**Authors:** Laura Scheffler, Constanze Sharapa, Tayyaba Amar, Andrea Buettner

**Affiliations:** ^1^Chair of Aroma and Smell Research, Department of Chemistry and Pharmacy, Emil Fischer Center, Friedrich-Alexander-Universität Erlangen-Nürnberg (FAU), Erlangen, Germany; ^2^Department Sensory Analytics, Fraunhofer Institute for Process Engineering and Packaging (IVV), Freising, Germany

**Keywords:** human milk, human urine, gas chromatography mass-spectrometry/olfactometry, allyl methyl sulfide, allyl methyl sulfoxide, allyl methyl sulfone, stable isotope dilution analysis (SIDA)

## Abstract

Ramson (Allium ursinum) is known for its typical garlic-like aroma. Both ramson and garlic belong to the genus allium which is characterized by a high content of sulfurous compounds. However, in contrast to garlic, ramson is in general not associated with an unpleasant breath following consumption. While there is data available regarding the metabolism of volatile garlic constituents in the human body, the metabolism of ramson was not yet addressed. To elucidate if ramson has an impact on the body odor, this study aimed at identifying volatile ramson-derived metabolites in human milk and urine. Therefore, milk and urine samples were gathered before and after ramson consumption, and were analyzed sensorially by a trained human sensory panel as well as chemo-analytically applying gas chromatography-mass spectrometry/olfactometry (GC-MS/O). Sensory evaluation revealed a garlic-/cabbage like odor in milk samples obtained after ramson consumption, demonstrating that ramson consumption affected the milk aroma. Analyzes by means of GC-MS/O further confirmed excretion of three ramson-derived metabolites in milk and urine samples collected after ramson consumption, namely allyl methyl sulfide (AMS), allyl methyl sulfoxide (AMSO) and allyl methyl sulfone (AMSO_2_). Of these metabolites only AMS had a garlic-/cabbage-like odor, while the other two were odorless. These metabolites were subsequently quantified using stable isotope dilution assays. Nine urine sets, each comprising eight urine samples, and nine milk sets, each comprising four samples, were analyzed. In case of the urine sets a time interval of about 24 h was monitored, in case of the milk sets a time interval of up to 9 h. Despite the fact that all samples contained the same metabolites there were relevant differences found between individual subjects, especially with regard to the temporal rate of metabolite excretion. Generally, the maxima of metabolite excretion were observed in milk sets within 3 h after ramson consumption. In urine the highest AMS and AMSO amounts were observed within 2 h whereas the maximum concentration of AMSO_2_ was reached about 2 to 4 h after ramson ingestion. This study suggests that ramson constituents are heavily metabolized in the human body.

## Introduction

Spice plants and condiments such as garlic are used world-wide to modulate the taste of dishes. However, this is associated with the side-effect that some people emit a distinct smell after consumption of garlic. The typical aroma of garlic is due to its pronounced content of sulfur-containing compounds. In general, a high sulfur substance content is typical for the genus Allium, which includes garlic. However, another representative of the genus Allium is ramson (Allium ursinum) which is commonly not associated with aversive effects like unpleasant breath despite the fact that the volatile oil of ramson comprises compounds that have also been identified in garlic, namely sulfides, ajoenes, and dithiins (Sendl et al., 1992; Benkeblia and Lanzotti, 2007; Godevac et al., 2008; Sabha et al., 2012). In relation to body odor and excretion processes of ramson-derived substances, biotransformation processes in the body need to be considered as these processes might lead to neoformation of compounds, whereas others might be degraded. Potential flavor effects of human nutrition on the sensory properties of human milk are in this context of special interest as human milk is commonly the sole food source of a newborn, at least during the first months of life. Besides possible effects on the infants' health, both positive and negative, it has been reported that the maternal diet can influence the suckling behavior and later dietary habits of the infant (Mennella et al., 2001). These behavioral effects have been attributed to a changed flavor profile of the milk, implicating that these flavor changes were supposed to be attractive to the children. However, final evidence in terms of sensory and chemo-analytical data, as well as proof of attractiveness of such flavor impressions to the infants was not provided. In this context it is interesting to note that intake of specific foods might impact diverse physiological processes and parameters such as blood pressure and secretory processes. There are several studies which demonstrate that the consumption of garlic can lead to reduction in blood pressure which is particularly distinctive in case of hypertensive patients (Reinhart et al., 2008; Ried, 2016; Choudhary et al., 2017). Such processes might also impact lactation processes, e.g., with elevated milk secretion, which might directly impact suckling behavior and milk intake of the children.

On the other hand, the analysis of urine opens the possibility to investigate low-molecular weight compounds. The dietary influence on the urine composition was demonstrated for instance for asparagus: people who consumed asparagus excreted urine with a sulfurous odor (Pelchat et al., 2011). Furthermore, the odorous compounds in urine have also been used as indicators for diseases or metabolic syndromes, e.g., trimethylaminuria, in which case the persons concerned excreted increased concentrations of trimethylamine via urine leading to a fishy odor (Humbert et al., 1970).

In case of garlic ingestion our group already identified three metabolites excreted via human milk and urine, namely allyl methyl sulfide (AMS), allyl methyl sulfoxide (AMSO) and allyl methyl sulfone (AMSO_2_) with only AMS present in garlic itself whereas the other two compounds are only formed within the human body (Scheffler et al., 2016a,b). In case of ramson there is no data available concerning its metabolism within the human body. To close this gap the present study aimed at elucidating volatile ramson-derived metabolites in humans. Therefore, human milk and urine samples gathered before and after ramson consumption were analyzed. Additionally, the identified compounds were quantified and their temporal excretion was monitored over a time period of about 9 h (milk) or 24 h (urine). The main consideration during all investigations was thereby to obey a consumption protocol that is well in line with a real-life ramson consumption scenario.

## Materials and methods

The materials and methods that were used in this study are in most parts in line with those described in our previous studies which addressed the identification and quantification of garlic-derived metabolites in human milk and urine (Scheffler et al., 2016a,b; Scheffler et al., submitted). For this reason only a short overview is provided here. Detailed descriptions are given in the respective studies and in the Supplementary Material provided for this study.

### Study design and ethics approval

The study was conducted in agreement with the Declaration of Helsinki. All participants gave written, informed consent prior to the testing day. They were able to withdraw from the study at any time. The study (registration no. 163_16 Bc) was approved by the Ethical Committee of the Medical Faculty, Friedrich-Alexander-Universität Erlangen-Nürnberg.

### Human milk samples

Human milk samples were obtained from 13 different mothers (age range 27–39 years, mean 33). The volunteers had no known illnesses and their milk production exceeded their infants' need. The sampling took place 9 to 37 seven weeks postpartum (mean 19 weeks). To avoid sulfurous compounds in the milk samples that were not associated with ramson consumption, the test persons were asked to avoid food containing high amounts of sulfur substances (e.g., garlic, onion, ramson, chives, cabbage, and leek) on the testing day and 2 preceding days. Additionally, they were asked to keep record of their food during these 3 testing days. Each mother provided one milk sample before ramson consumption and three further samples after ramson ingestion. The samples were collected according to the normal lactation period of each mother and immediately analyzed. Four consecutive milk samples formed a milk set in each case. In a pre-test one mother consumed about 3 g of ramson. All other test persons consumed about 10 g of ramson.

Four milk sets were collected for identification experiments. They were labeled “*M*” with Arabic numerals, e.g., *M 2-1* to *M 2-4*. Quantification experiments were performed after completion of identification experiments. In case of the quantification experiments nine milk sets were gathered and labeled “*M*” with Roman numerals, e.g., M *II-1* to *M II-4*.

### Human urine samples

Human urine samples were obtained from four volunteers (age 24–28 years, mean 26, two females, two males). One volunteer conducted the whole experimental series four times, the other three volunteers participated twice. Urine samples were collected in sterile brown glass bottles. At the testing day as well as 2 days preceding the testing day, the test persons were instructed to avoid sulfurous compounds and to record consumed foods and beverages as described for the milk sampling (cf. chapter 2.2). On the testing day ramson was freshly washed, ground and aliquoted into portions of 10 g. The volunteers provided one urine sample before and seven samples after ingestion of 10 g of ramson at about the following times: 0.5, 1, 2, 4, 6, 8, and 24 h after ramson consumption. Eight consecutive urine samples comprised one urine set. The samples were kept frozen at −80°C until further analysis.

To rule out illnesses of the test persons, the first sample of each set was tested with a dipstick test. With the multiple test stripes (Combi-Screen Plus, Analyticon Biotechnologies AG, Lichtenfels, Germany) simultaneous testing of following urine parameters was possible: ascorbic acid, bilirubin, blood, glucose, ketones, leucocytes, nitrite, pH, protein, specific gravity/density, and urobilinogen.

For identification experiments one urine set was collected. The respective samples were labeled *U 1-1* to *U 1-8*. For quantification analyses nine urine sets were analyzed. The urine samples of each set were labeled “*U*” with Roman numerals (e.g., *U II-1* to *U II-8*) and according to their sampling time:

- Pre: 3 to 7 min before ramson consumption
- 0.5 h post: 0.45 h to 0.55 h after ramson consumption
- 1 h post: 1.00 h to 1.05 h after ramson consumption
- 2 h post: 1.95 h to 2.10 h after ramson consumption
- 4 h post: 4.00 h to 4.10 h after ramson consumption
- 6 h post: 6.00 h to 6.10 h after ramson consumption
- 8 h post: 8.00 h to 8.15 h after ramson consumption
- 24 h post: 23.95 h to 24.20 h after ramson consumption

*U II* to *U V* were obtained at the same testing day with each test person consuming ramson portions of about 10 g. Likewise, *U VI* to *U IX* were provided at the same testing days.

### Isolation, identification and quantification of ramson-derived metabolites

The volatile fraction of the urine and milk samples was isolated by means of Solvent-assisted flavor evaporation (SAFE)-distillation (Engel et al., 1999). The obtained distillate was analyzed by means of high resolution gas chromatography-olfactometry (HRGC-O) as well as high resolution gas chromatography-mass spectrometry (HRGC-MS) and two-dimensional HRGC-MS/O (heart-cut). Ramson-derived metabolites were identified by comparing their retention indices (RI) of two analytical capillaries with different polarities (DB-5 and DB-FFAP), their respective odor as well as their mass spectra with those of reference standards. RI values were calculated according to van Den Dool and Kratz (1963). A relative concentration of the identified metabolites was calculated by normalizing respective peak areas to the amount of investigated milk or urine (in kg) in order to express the concentration in units of area/kg milk or urine. In case of the urine samples, the concentration was additionally expressed as area/mmol creatinine. Therefore, the creatinine levels in each urine sample were determined using a creatinine kit (Labor+Technik Eberhard Lehmann GmbH, Berlin, Germany). The peak area was divided by the amount of investigated urine (in L) and the creatinine content (in mmol/L) of the respective urine sample. In subsequent studies the respective metabolites were quantified by means of stable isotope dilution analysis (SIDA). Isotopically labeled standards were added to the samples prior to sample work up and solutions consisting of defined mixtures of analyte standards and their respective isotopically labeled standards were analyzed. Calibration curves were calculated as functions between the intensity ratios of standard to labeled standard and their respective mass ratios (cf. Table 1). Calibration curves were prepared in triplicates, and at 3 different days. For quantification the average of these calibration curves was used. With the resulting calibration function, the known amount of isotopic labeled standard added to the sample and the intensity ratio of analyte to isotopic labeled standard the amount of the analyte in the sample was calculated.

**Table 1:** Parameters for quantification including the instrument which was used for measurement, selected m/z-ratios of analytes and isotopically labeled standards and calibration factors.

	**Instrument**	**Analyte**		**Labeled standard**		**Calibration function**	***R*^2^**
		**Compound**	**m/z**	**Compound**	**m/z**		
Milk	GC-GC-MS	AMS	88	^2^H_3_- AMS	91	y = 0.9171x – 0.0492	0.9981
	GC	AMSO	104	^2^H_3_- AMSO	107	y = 1.0106x + 0.0045	1.0000
	GC	AMSO_2_	120	^2^H_3_- AMSO_2_	123	y = 0.9727x + 0.0058	0.9999
Urine	GC-GC-MS	AMS	88	^2^H_3_- AMS	91	y = 0.9289x – 0.029	0.9996
	GC	AMSO	104	^2^H_3_- AMSO	107	y = 1.0034x + 0.0081	1.0000
	GC	AMSO_2_	120	^2^H_3_- AMSO_2_	123	y = 0.9746x + 0.008	0.9999

In order to express the concentration of ramson-derived metabolites as μg/kg milk or urine or μg/mmol creatinine, the calculated amounts of AMS, AMSO and AMSO_2_ were divided by the amount of investigated sample (in kg) or normalized to creatinine content as described above.

### Aroma profile analysis of human milk samples

Milk samples were additionally evaluated sensorially (orthonasally) prior to the sample work up. Aroma profile analysis was performed based on sensory pre-evaluations and the intensity of the following attributes were rated on a scale from 0 (no perception) to 3 (strong perception): hay-like, fishy, fatty, rancid, sweaty, metallic, grassy-green, sweet, egg white-like, buttery, lactic, and garlic/cabbage-like. The evaluated attributes corresponded to those described in Scheffler et al. (2016b).

## Results

### Aroma profile analysis of human milk samples in relation to ramson ingestion

**Figure 1:**
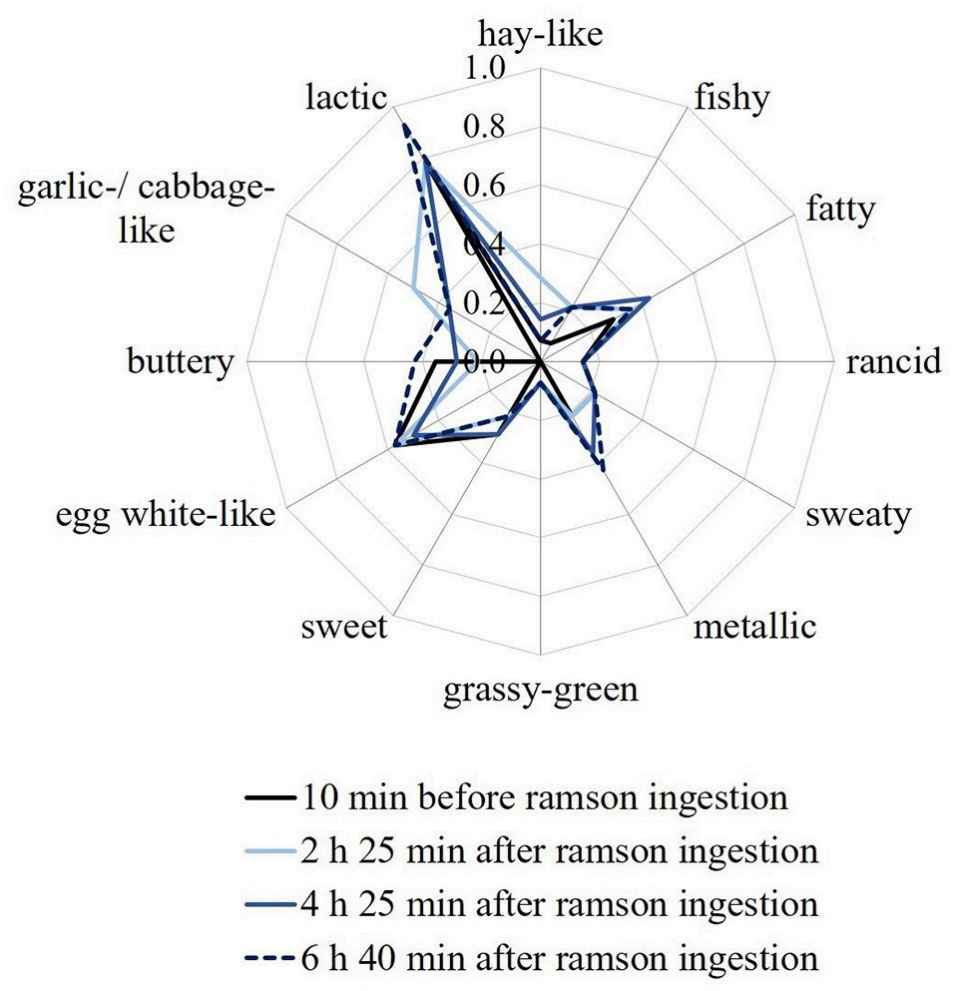
Odor profiles of human milk samples of set *M 2*, as a representative sample. The samples were collected at different times before and after ingestion of 10 g of ramson: 10 min before, 2 h 25 min after, 4 h 25 min after and 6 h 40 min after ramson intervention. Panelists were asked to rate the orthonasal perception on a scale from 0 (no perception) to 3 (strong perception). Values are mean ratings of all panelists. Note: The scale is only presented up to the value of 1 for better visualization.

Aroma profile analyses were performed on all human milk samples that had been obtained before and after ramson consumption in the course of identification experiments. Samples were rated according to the attributes fishy, fatty, metallic, grassy-green, rancid, sweaty, buttery, sweet, hay-like, egg white-like, lactic, and garlic-/cabbage-like. The overall intensity of the milk samples as well as the odor attributes were mostly rated as being just detectable (intensity 1) or even not perceivable (intensity 0). Overall, the attribute garlic-/cabbage-like was not perceivable in all samples obtained before ramson intervention. After consumption of 10 g of fresh ramson the milk samples were rated as having a slight (0.5–1.0) garlic-/cabbage-like odor by most of the panelists. The odor change was most pronounced in milk samples that were provided 2 to 5 h after ramson consumption. Exemplary aroma profiles of a sample series of one milk set are displayed in Figure 1. Nevertheless, this does not rule out a potential sensory detection via tasting that was, however, not possible due to work safety considerations. All other aroma profiles of the milk samples that were also collected for the identification experiments are provided in Figure S1 of the Supplementary Material. Moreover, aroma profile analyses were repeated for those samples that were collected during the quantification experiments. Also in these cases a slight garlic-/cabbage-like odor was perceived in the milk samples that were obtained after ramson consumption (data not shown). Hence, the results from the identification experiments could be confirmed.

### Identification of ramson-derived metabolites

#### Comparative GC-O analysis

Comparative GC-O analyses were performed for the corresponding human milk and urine samples collected before and after ramson consumption in order to identify odor-active compounds that derived from ramson consumption. In both, urine and milk samples, the analyses revealed one additional odor-active substance that was only detectable in samples obtained after ramson intervention. This substance had a garlic-/cabbage-like odor, a RI < 1000 on the FFAP capillary and RI 702 on the DB-5 capillary. By comparison of the odor quality and the respective RI-values with reference substances (cf. Table 2), this compound was identified as AMS. All other odors were perceived in all samples, irrespective of ramson consumption, hence they cannot be associated with ramson consumption but rather contribute to the typical smell of human milk and urine.

**Table 2:** Compilation of investigated substances, their chemical structures, retention indices (RI) on two different chromatographic capillaries (DB-FFAP and DB-5), their odor qualities and literature reports on these substances.

**Substance (abbreviation)**	**Structure**	**RI**		**Odor quality**	**Detected in**				**Previously detected in**
		**FFAP**	**DB-5**		**M pre**	**M after**	**U pre**	**U after**	
2-Vinyl-4H-1,3-dithiin	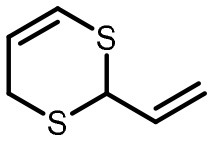	1767	1225	Garlic-like	n.d.	n.d.	n.d.	n.d.	Garlic^a−g^ Ramson^h^
3-Vinyl-4H-1,2-dithiin	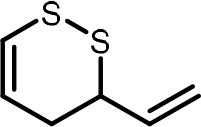	1671	1198	Pungent, garlic-like	n.d.	n.d.	n.d.	n.d.	Garlic^a−c, e, g, i, j^ Ramson^h^
Allyl methyl disulfide (AMDS)	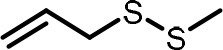	1206	918	Cooked garlic-like	n.d.	n.d.	n.d.	n.d.	Garlic^a−c, f, g, i, j^ Ramson^h, k−n^ Human breath after garlic consumption^o−s^
Allyl methyl sulfide (AMS)	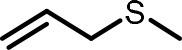	< 1,000	702	Garlic-/cabbage-like	n.d.	yes	n.d.	Yes	Garlic^a, d, f, i^ Ramson^k, m^ Human breath^o−v^, human milk^w^, human urine^x^ after garlic consumption
Allyl methyl sulfone(AMSO_2_)	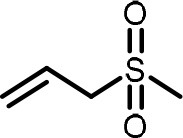	1917	1058	Odorless	n.d.	Yes	n.d.	Yes	Human milk^w^, human urine^x^ after garlic consumption
Allyl methyl sulfoxide (AMSO)	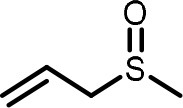	1717	1003	Odorless	n.d.	Yes	n.d.	Yes	Human milk^w^, human urine^x^ after garlic consumption
Allyl propyl disulfide (APDS)	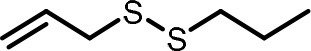	1360	1097	Garlic-like	n.d.	n.d.	n.d.	n.d.	Garlic^a−c, i^ Ramson^h, k−m^
Allyl propyl sulfide (APS)	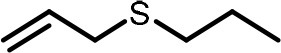	1051	876	Garlic-/onion-like	n.d.	n.d.	n.d.	n.d.	Ramson^m^
Diallyl disulfide (DADS)	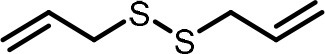	1404	1083	Garlic-like	n.d.	n.d.	n.d.	n.d.	Garlic^a−g, i, j^ Ramson^h, k−n^ Human breath after garlic consumption^o−v^
Diallyl sulfide (DAS)	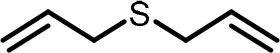	1085	863	Garlic-like	n.d.	n.d.	n.d.	n.d.	Garlic^a, b, d, f, g, i, j^ Ramson^k, m, n^ Human breath after garlic consumption^o, u, s^
Diallyl sulfone (DASO_2_)	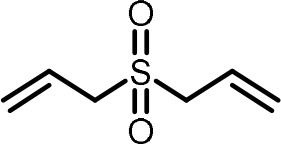	2018	1193	Odorless	n.d.	n.d.	n.d.	n.d.	Rat liver, blood and urine after treatment with DAS^y^
Diallyl sulfoxide (DASO)	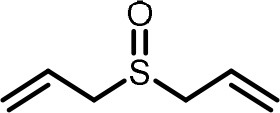	1858	1159	Garlic-like	n.d.	n.d.	n.d.	n.d.	Rat liver, blood and urine after treatment with DAS^y^
Diallyl trisulfide (DATS)	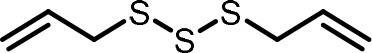	1712	1310	Garlic-like	n.d.	n.d.	n.d.	n.d.	Garlic^a−g, i, j^ Ramson^h, l−n^ Human breath after garlic consumption^o, s^
Dimethyl disulfide (DMDS)	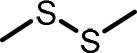	1018	739	Cabbage-like	n.d.	n.d.	n.d.	n.d.	Garlic^a−d, f, g, i, j^ Ramson^h, k, m, n^ Human breath after garlic consumption^o^
Dimethyl trisulfide (DMTS)	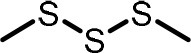	1295	972	Cabbage-like	n.d.	n.d.	n.d.	n.d.	Garlic^a, b, f, g, i^ Ramson^h, k−n^
Dipropyl disulfide (DPDS)	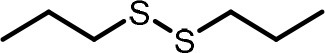	1312	1111	Garlic-like	n.d.	n.d.	n.d.	n.d.	Ramson^k, m^
Dipropyl trisulfide (DPTS)	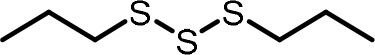	1603	1337	Cooked garlic-/cabbage-like	n.d.	n.d.	n.d.	n.d.	Ramson^h, k, m^
Methyl propyl disulfide (MPDS)	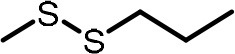	1168	935	Garlic-/cabbage-like	n.d.	n.d.	n.d.	n.d.	Garlic^a, c, d, f, g, j^ Ramson^k−n^
Methyl propyl trisulfide (MPTS)	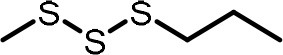	1457	972	Garlic-/cabbage-like	n.d.	n.d.	n.d.	n.d.	Ramson^k−m^

*The presence of the target compounds in milk and urine samples collected before (M 2-1 and U 1-1) and after (M 2-2 and U 1-4) consumption of 10 g of raw ramson is provided. Confirmation/ identification or exclusion of the compounds was conducted by means of HRGC-GC-MS. M 2-2 (2.4 h after ramson consumption) and U 1-4 (2 h after ramson consumption) are displayed as representative examples of data obtained for the samples provided after ramson consumption*.

*^a^(Yu et al., 1989), ^b^(Mazza et al., 1992), ^c^(Mondy et al., 2001), ^d^(Lee et al., 2003), ^e^(Abu-Lafi et al., 2004), ^f^(Calvo-Gómez et al., 2004), ^g^(Kimbaris et al., 2006), ^h^(Sobolewska et al., 2013), ^i^(Tokarska and Karwowska, 1983), ^j^(Galoburda et al., 2013), ^k^(Schmitt et al., 2005), ^l^(Godevac et al., 2008), ^m^(Radulović et al., 2015), ^n^(Blazewicz-Wozniak and Michowska, 2011), ^o^(Cai et al., 1995), ^p^(Hansanugrum and Barringer, 2010), ^q^(Munch and Barringer, 2014), ^r^(Suarez et al., 1999), ^s^(Taucher et al., 1996), ^t^(Buhr et al., 2009), ^u^(Rosen et al., 2000), ^v^(Lawson and Wang, 2005), ^w^(Scheffler et al., 2016b), ^x^(Scheffler et al., 2016a), ^y^(Brady et al., 1991), n.d.: not detected*.

#### Identification of ramson-derived volatiles in milk and urine samples applying HRGC-MS and HRGC-GC-MS

**Figure 2:**
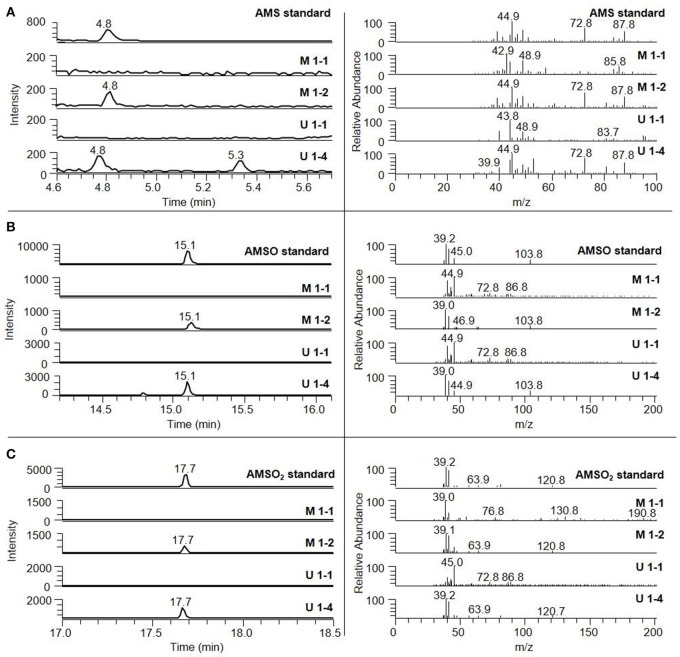
Identification of ramson-derived components in human milk and human urine. The milk samples were collected 10 min before (*M 2-1*) and 2 h 25 min (*M 2-2*) after ramson consumption. Urine samples were gathered 5 min before (*U 1-1*) and 2 h (*U 1-4*) after ramson consumption. **(A)** AMS, **(B)** AMSO, **(C)** AMSO_2_. The respective mass spectra are shown on the right side; they correspond to the elution time of the respective standard compound.

In order to further identify less odor-active or odorless ramson-derived compounds in the milk and urine samples, the chromatograms obtained from GC-MS analyses of control samples prior to ramson intervention were compared to those obtained after intervention in full scan-mode. Additionally, the samples were screened for potential ramson-derived metabolites via targeted search, taking into consideration compounds that had previously been reported in literature as constituents of ramson. Moreover, we screened for substances that had been reported in garlic, or in breath after garlic consumption, respectively, or that had been proposed as potential metabolites in humans based on animal studies (cf. Table 2). To this aim, a targeted search by means of GC-MS and GC-GC-MS was conducted utilizing standard solutions of the respective compounds. GC-GC-MS analyses thereby allowed screening for minor quantities of the target substances in the low μg/mL-range. The concentrations of the respective reference compounds thereby ranged between 0.5 and 12.7 μg/mL DCM. These investigations revealed two additional ramson-derived metabolites, namely AMSO and AMSO_2_. Moreover, GC-GC-MS analysis confirmed the identification of AMS in urine and milk samples collected after ramson consumption. Representative chromatograms are displayed in Figure 2. The other compounds of the targeted search were not detectable in any urine or milk samples, even at concentrations as low as about 1–10 μg/mL, irrespective if sampling took place prior to or after ramson ingestion. These compounds were: 2-vinyl-4H-1,3-dithiin, 3-vinyl-4H-1,2-dithiin, allyl methyl disulfide (AMDS), allyl propyl disulfide (APDS), allyl propyl (APS), diallyl disulfide (DADS), diallyl sulfide (DAS), diallyl sulfoxide (DASO_2_), diallyl sulfone (DASO), diallyl trisulfide (DATS), dimethyl disulfide (DMDS), dimethyl trisulfide (DMTS), dipropyl disulfide (DPDS), dipropyl trisulfide (DPTS), methyl propyl disulfide (MPDS), and methyl propyl sulfide (MPTS).

### Quantification and time dependency of excretion of ramson-derived metabolites

#### Quantitative analysis of ramson-derived metabolites in human milk

The quantification of ramson-derived metabolites in human milk was carried out on nine milk sets, each comprising four milk samples. The exact sampling times as well as volume and weight of the samples are provided in Table S1.

**Figure 3:**
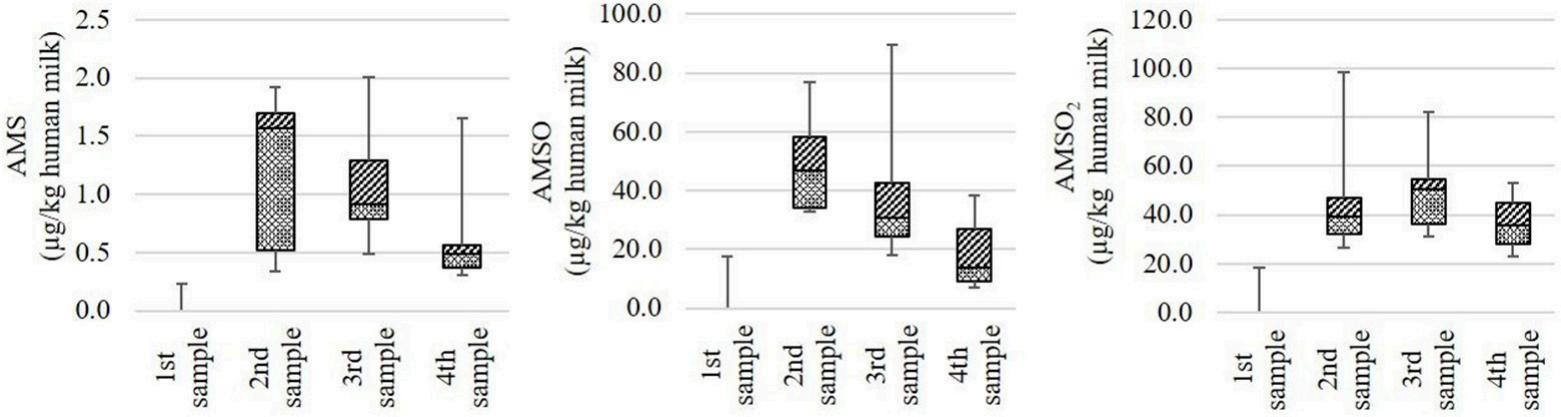
Box plot (mean value, markers at minimum and maximum metabolite concentration, box perc. 25–75%) of concentrations of ramson metabolites (AMS, AMSO, and AMSO_2_) in human milk, expressed as μg/kg human milk. Data comprise nine milk sets each being composed of four milk samples: one sample was collected before (“1st sample”) and three samples after (“2nd sample” to “4th sample”) ramson ingestion.

In the majority of cases the ramson-derived metabolites AMS, AMSO, and AMSO_2_ were only detected in those milk samples that were obtained after ramson consumption. Only in case of milk *set V* and *VI* small amounts of these metabolites were observed in the samples gathered prior to ramson consumption. According to the recorded food protocols, the respective test persons consumed bratwurst and kohlrabi 1 day prior to the respective testing days. Bratwurst can comprise multiple spices, amongst others garlic. Garlic, as well as kohlrabi are rich in sulfur-containing compounds, which might explain the detected metabolites in the samples collected prior to ramson consumption. Despite these small amounts in the control samples, distinct increases in the metabolite concentrations were observed in the subsequently collected milk samples. The highest observed AMS concentrations in milk samples obtained after ramson consumption were found to be quite consistent, with values in the range between 1.7 and 2.0 μg/kg milk. AMSO and AMSO_2_ reached values between 38.4 and 89.6 μg/kg milk and 53.0 and 98.6 μg/kg milk, respectively. Commonly, the maxima were either detected in the first or in the second milk sample after ramson consumption which corresponds to a time interval between 1.5 to 5 h after ramson ingestion. Thereby, the AMSO maximum tended to appear slightly earlier than the AMSO_2_ maximum. In most cases the highest concentrations of the metabolites AMS and AMSO were reached within 3 h after ramson consumption (as was the case for seven out of nine milk sets). The highest amount of AMSO_2_ was mostly observed between 3 and 5 h (as was the case for six out of nine milk sets). Afterwards, a decline in metabolite concentration was observed which was pronounced in case of AMSO but slow in case of AMSO_2_. This trend is visualized in Figure 3.

The metabolite concentrations for each milk sample, arranged according to milk sets, are provided in Table S1.

**Figure 4:**
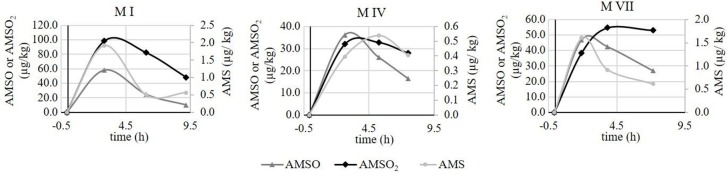
Time-resolved metabolite profiles of AMS, AMSO and AMSO_2_. Profiles are exemplarily shown for milk sets *M I, M IV*, and *M VII*. • AMS,▴ AMSO, ♦ AMSO_2_. Time 0 h corresponds to the time of ramson consumption.

The temporal monitoring of the excreted metabolite concentrations revealed some inter-individual variation between test persons. For better visualization, representative time-resolved metabolite profiles are displayed in Figure 4.

Generally, differences were observed both in the excreted metabolite concentrations, as described above, as well as in the time dependency of excretion. It is important to note that, however, all test persons consumed the same amount of fresh ramson following the same consumption protocol. The time-resolved metabolite profiles revealed that for some milk samples the maxima of all three metabolites coincided in the first milk sample gathered after ramson consumption (e.g., *M I*). On the other hand, there were also milk sets where the maximum of AMSO_2_ was detected after the maximum of AMSO (e.g., *M VII*), and other cases, where the maximum of AMS appeared in the milk sample following the maximum of AMSO (e.g., *M IV*). To further clarify whether the maxima of the metabolites coincide or are excreted with a time delay, a larger sample set should be analyzed with shorter time intervals between the samples. However, the present study aimed to investigate the concentrations of ramson-derived metabolites in human milk in a real-life situation which included that the mothers kept their normal feeding habits.

#### Quantitative analysis of ramson-derived metabolites in human urine

The quantification of ramson-derived metabolites in human urine was likewise carried out on nine different urine sets, each comprising eight urine samples. The exact sampling times as well as volume, weight and creatinine content of each sample are provided in Table S2.

**Figure 5:**
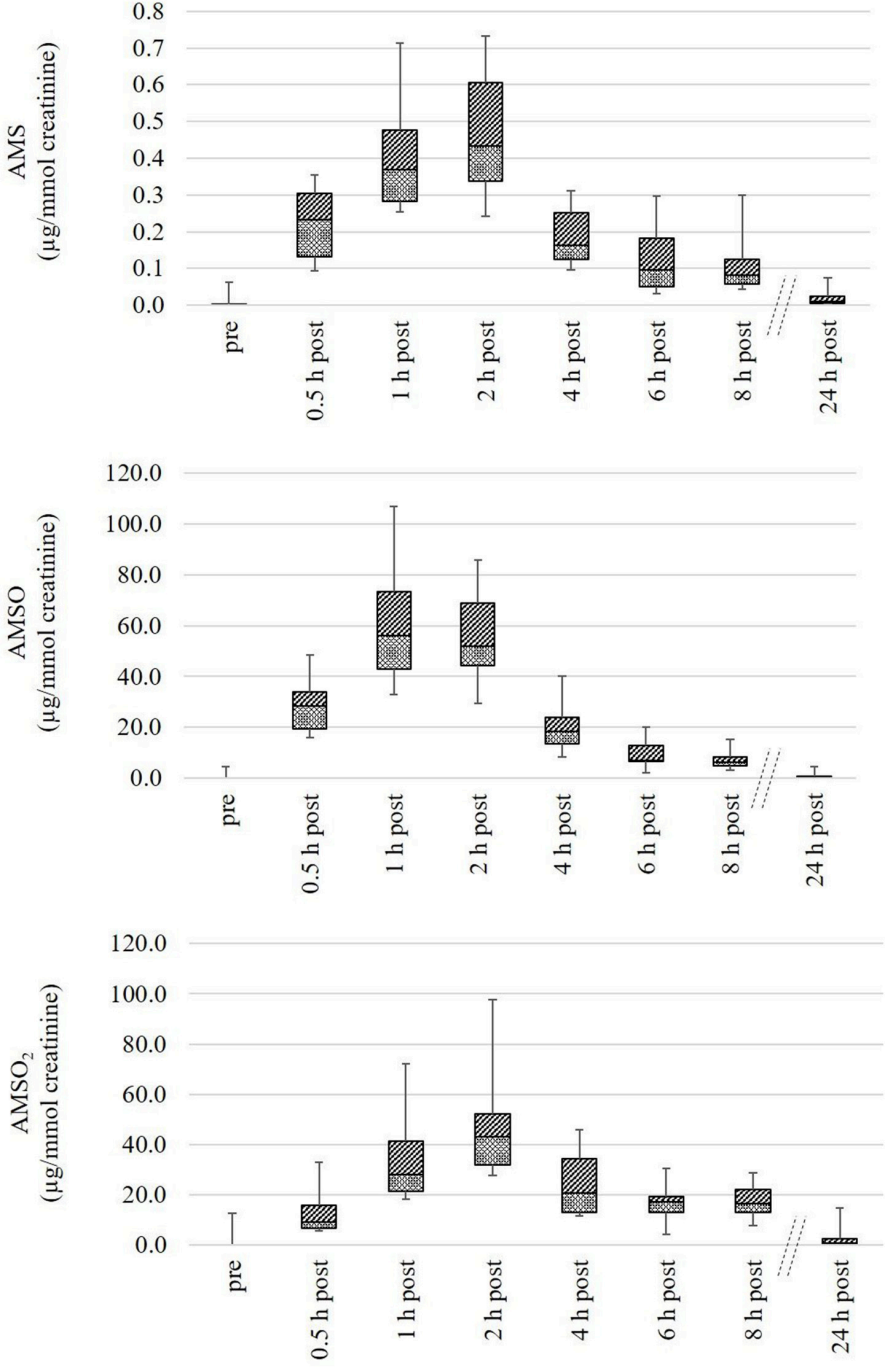
Box plot (mean value, markers at minimum and maximum metabolite concentration, box perc. 25–75%) of concentrations of ramson metabolites (AMS, AMSO, and AMSO_2_) in human urine, expressed as μg/mmol creatinine. Data comprise nine urine sets each being composed of eight urine samples: one sample was collected before (“pre”) and seven samples after (“0.5 h post” to “24 h post”) ramson ingestion.

The metabolites AMS, AMSO, and AMSO_2_ were only detected in urine samples gathered after ramson consumption with the sole exception of set *U IX*. In this urine set small amounts of the respective metabolites were detected in the sample collected before ramson intervention. According to the food protocol the respective test person consumed chicken escalope with mustard the day prior to the experiment. Mustard is rich in glucosinolates (Belitz et al., 2008), compounds that are sulfur-containing and could therefore be responsible for the respective derivatives in the first urine sample. Although the presence of AMS, AMSO, and AMSO_2_ could not be excluded in this case, distinct increases were observed after ramson consumption. Likewise, AMS, AMSO, and AMSO_2_ showed a rapid onset in all other urine sets after ramson consumption. The maximum AMS concentrations in the respective urine sets were found to be in the range between 0.4 and 0.9 μg/kg urine, whereas the maximum AMSO and AMSO_2_ concentrations ranged between 46.3 and 81.2 μg/kg urine and 40.8 and 87.1 μg/kg urine, respectively. However, relative metabolite concentrations in urine strongly depend on the water intake of the test persons. This discontinuous variability can be taken account of by normalizing the metabolite concentrations to the creatinine content of the respective urine sample. As a result, urine samples within one urine set as well as urine sets from different test persons can be compared with each other, and allow a better comparison of the temporal excretion processes in different subjects. Overall, the highest normalized AMS and AMSO concentrations were observed between 1 and 2 h after ramson consumption, and varied, in case of AMS, between 0.3 and 0.7 μg/mmol creatinine, and, in case of AMSO between 32.9 and 106.9 μg/mmol creatinine. The highest amounts of AMSO_2_ in urine generally varied between 27.5 and 97.7 μg/mmol creatinine. The AMSO_2_ maximum was either detected temporally coinciding with the AMSO maximum (as was the case for four urine sets) or with a temporal delay of about 1 to 2 h (as was the case for five urine sets). In most cases the AMSO_2_ maxima were observed about 2 h after ramson consumption. Only in case of *U IX*, a maximum was observed at 1 h, and in case of *U VII* at 4 h after ramson consumption. A high concentration of AMSO_2_ commonly correlated with high amount of AMSO and AMS in either case. In some urine sets a second increase in metabolite concentration could be observed about 6 to 8 h after ramson consumption (e.g., *U III* and *U V*). However, this second increase was not as distinct as the first one, commonly reaching about 10–70% of the total intensity of the first maximum. In Figure 5 the quantitative analyses of all urine samples are summarized as a box plot diagram. The metabolite concentrations for each urine sample, arranged according to urine sets, are additionally provided in Table S2.

**Figure 6:**
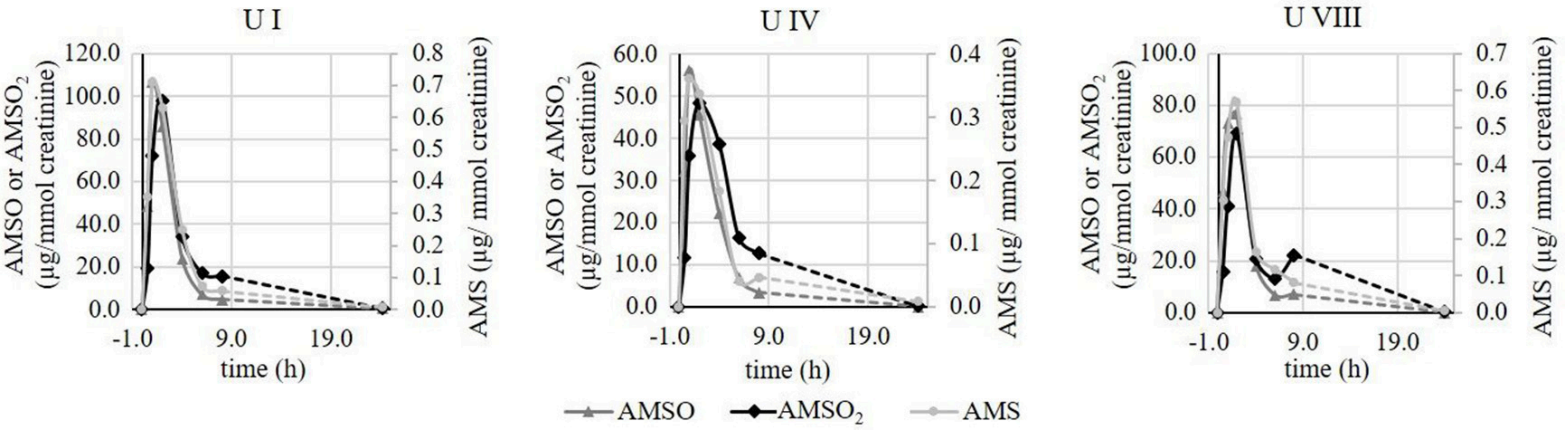
Time-resolved metabolite profiles of ramson-derived metabolites (AMS, AMSO, and AMSO_2_) of three urine sets (*U I, U IV*, and *U VIII*) provided by the same test person at different days. Time 0 h corresponds to the time of ramson consumption. • AMS, ▴ AMSO, ♦ AMSO_2_. Broken line: time interval, when no sample was collected.

We additionally observed some variance in the metabolite excretion patterns for the same individuals when test persons provided urine sets at different days. In Figure 6, time-resolved metabolite profiles of three urine sets, (*U I, U IV*, and *U VIII*) provided by the same test person, are exemplarily displayed. The temporal excretion pattern between the testing days was comparable; in each case the highest metabolites concentrations were observed between 1 and 2 h for each of the investigated metabolites. However, the amounts of excreted metabolites differed between the testing days. In *U I* the maximal concentration of AMSO and AMSO_2_ was about 100 μg/mmol creatinine (106.9 and 97.7 μg/mmol creatinine, respectively). In *U IV* the highest amount of these metabolites was about 50 μg/mmol creatinine (56.2 and 48.3 μg/mmol creatinine) and in *U VIII* a maximum concentration of 76.6 μg/mmol creatinine AMSO and 69.1 μg/mmol creatinine AMSO_2_ was detected. Likewise, the maximum concentration of AMS differed between the urine sets, albeit to a lesser extent: AMS concentrations of 0.7, 0.4 and 0.6 μg/mmol creatinine were observed in *U I, U IV*, and *U VIII*, respectively.

**Figure 7:**
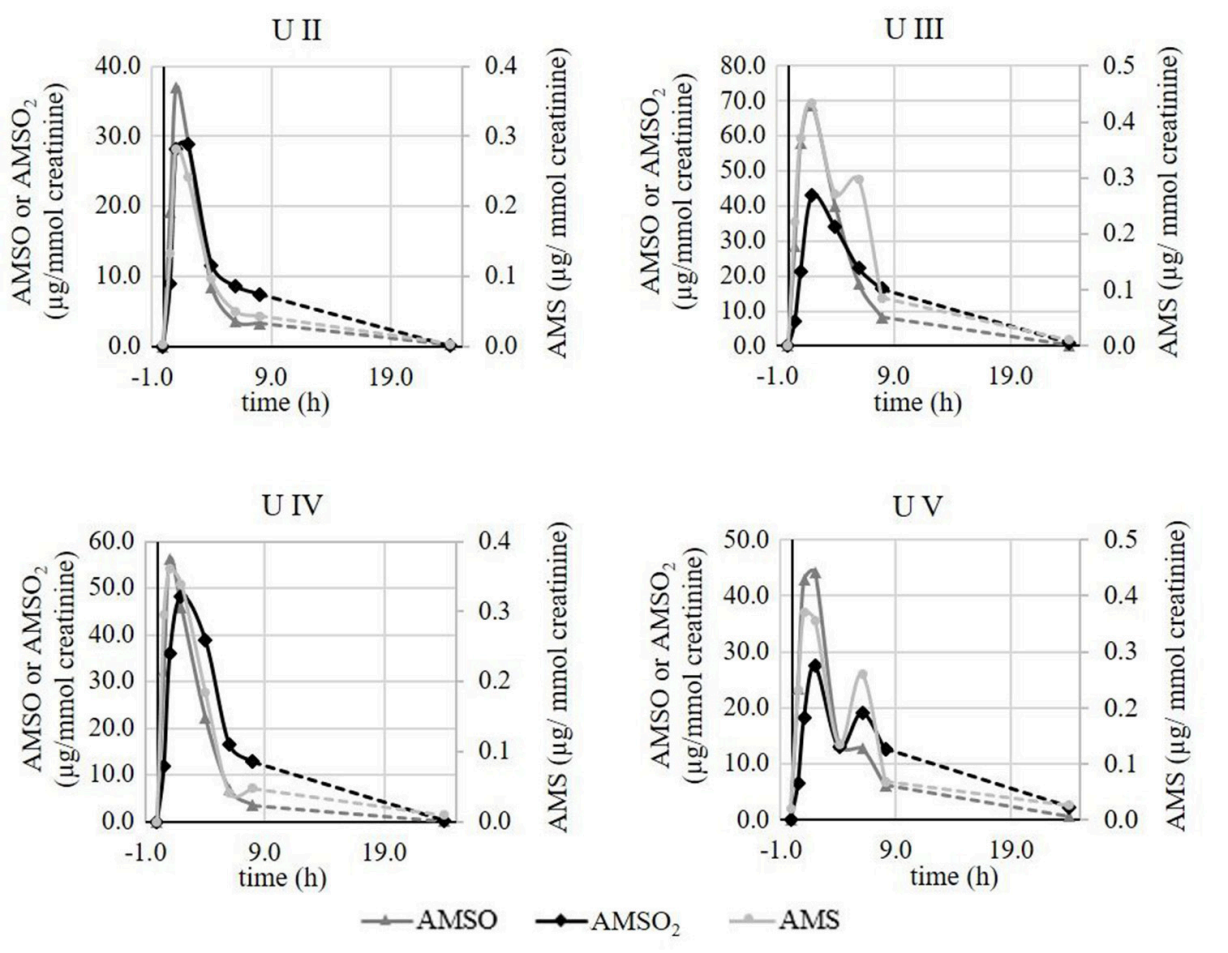
Time-resolved metabolite profiles of ramson-derived metabolites (AMS, AMSO, and AMSO_2_) of four urine sets (*U II, U III, U IV, U V*). All four test persons consumed ramson aliquots from the same ramson sample. Time 0 h corresponds to the time of ramson consumption. • AMS, ▴ AMSO, ♦ AMSO_2_. Broken line: time interval, when no sample was collected.

To exclude that these differences were due to different ramson samples and correspondingly natural differences in the respective plant material, additional experiments were conducted testing a total of four subjects on the same day. Panelists were asked to consume the same aliquots of the same ramson sample. The time-resolved metabolite profiles of the respective test persons obtained during this testing day were recorded and data are shown in Figure 7.

The analyses revealed that there were still differences in the respective metabolite profiles, although all four test persons consumed 10 g aliquots from the same ramson sample. All profiles revealed a metabolite increase within 2 h after ramson consumption. However, in case of test *person V* a second increase was observed after about 6 h for all three ramson-derived metabolites. Likewise, a second increase was monitored in urine set *U III* for AMS. In all other cases we observed only one maximum. Furthermore, there were differences in the metabolite concentrations excreted after ramson consumption comparable to those that were observed when subjects were tested at different days. While the maximum AMS concentration showed only minor differences, with values between 0.3 and 0.4 μg/mmol creatinine, AMSO maxima varied between 36.9 and 68.8 μg/mmol creatinine and in case of AMSO_2_ between 27.5 and 48.3 μg/mmol creatinine. These observations are limited to four test persons. It provides a first insight into the metabolism and excretion processes of ramson-derived metabolites. However, the differences between test persons might be more or less pronounced if a larger test group is tested.

## Discussion

Aroma profile analyses, conducted via orthonasal evaluation, revealed a change in the human milk odor after consumption of about 10 g of ramson which was most pronounced about 2 to 5 h after ramson consumption. As confirmed by our expert sensory panel this change comprised a garlic-/cabbage-like odor which was only perceived in milk samples collected after ramson consumption. Such change was already described in milk samples that were obtained after garlic consumption (Scheffler et al., 2016b). However, whereas a clear aroma change was already observed after consumption of 3 g of raw garlic, in case of ramson about 10 g had to be consumed. Hence, garlic seems to have a higher impact on the human body odor compared to ramson. This is in line with the general observation that garlic consumption influences the body odor, e.g., breath, to a higher extent than ramson consumption (Borrelli et al., 2007). However, it still needs to be kept in mind, that all observed sensory attributes, including the attribute garlic-/cabbage-like, were rated in an orthonasal evaluation as being just detectable (intensity 1 or lower). Nevertheless, this does not rule out that the effect might have been more pronounced during tasting, i.e., retronasal evaluation. However, due to work safety consideration this type of evaluation was not carried out by the expert panel.

In relation to ramson consumption three metabolites were identified in human urine and milk, namely AMS, AMSO, and AMSO_2_. Of these, AMS had a garlic-/cabbage-like odor whereas the other two compounds were odorless. We found that AMS is related to the aroma change in the milk samples after ramson consumption.

Generally, the transfer of food components originating from the maternal diet into breast milk are not yet fully understood but it has been postulated that compounds that are relevant to the specific flavors of foods and beverages may affect the sensory quality of breast milk, as has been reported in the case of carrots or alcohol (Mennella and Beauchamp, 1991, 1999). On the other hand, studies on the potential influence of herbal tea or encapsulated fish oil products contradict these findings, as they ruled out any sensory or chemical changes in human milk composition (Sandgruber et al., 2011; Denzer et al., 2015). Here, we report a condiment that has the potential of affecting the milk composition of nursing mothers. In most cases, irrespective of the consumed amount of ramson and the investigated excretion product, AMS, AMSO, and AMSO_2_, were only detected in samples after ramson consumption, demonstrating that they clearly derived from ramson consumption. In previous studies we were able to identify the same metabolites in urine and milk samples obtained after garlic consumption (Scheffler et al., 2016a,b). It can be assumed that AMS, AMSO, and AMSO_2_ are not ramson-specific metabolites but may be rather linked to several sulfur constituents-bearing foods. On the other hand, further sulfury and/or odorous compounds of ramson such as 2-vinyl-4H-1,3-dithiin, 3-vinyl-4H-1,2-dithiin, AMDS, APDS, APS, DADS, DAS, DATS, DMDS, DMS, DPDS, DPTS, MPDS, and MPTS were proven to be absent at levels as low as 1–10 μg/mL, both in samples collected before as well as after ramson consumption. This leads to the conclusion that ramson constituents are heavily metabolized and eliminated via other physiological routes.

**Figure 8:**
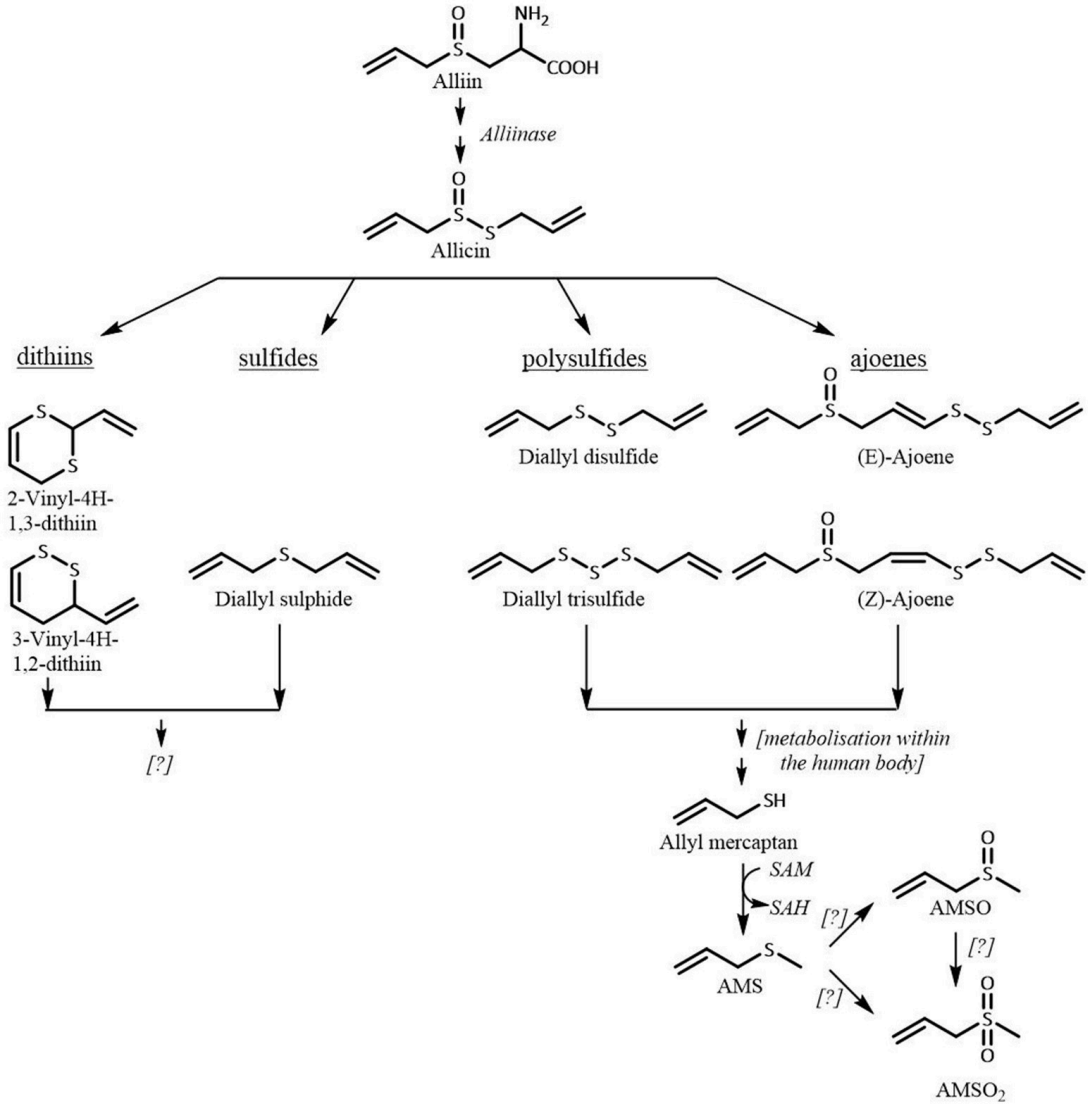
Metabolic fate of alliin. SAM, S-adenosylmethionine; SAH, S-adenosylhomocysteine.

The highest observed AMSO concentration in human milk ranged between 38 and 90 μg/mL human milk; the highest observed AMSO_2_ concentration ranged between 53 and 99 μg/mL human milk. Similar concentrations of the respective metabolites were observed in human milk after garlic consumption. However, the amount of consumed ramson was more than three times as high as the consumed garlic amount (10 vs. 3 g) (Scheffler et al., 2018). The same applies when ramson- and garlic-derived metabolites in urine are compared. These differences are likely related to the different compositions of garlic and ramson. Both plants contain high amounts of S-alk(en)ylcysteine sulfoxides. The quantitatively dominating S-alk(en)ylcystein sulfoxide in garlic is alliin [S-allylcysteine sulfoxide, 481–1,140 mg/100 g fresh weight (fw)] followed by methiin (S-allylcysteine sulfoxide 50–126 mg/100 g fw) (Kubec et al., 1999) whereas in ramson it is methiin (60 mg/100 g fw) followed by alliin (40 mg/100 g fw) (Kubec et al., 2000). Accordingly, garlic and ramson comprise the same sulfurous compounds that are odorless themselves, but are precursors for odor-active compounds. These precursors are present at different ratios and concentrations, with garlic containing about ten times the quantity of S-alk(en)ylcysteine sulfoxides compared to ramson. Upon rapture various aroma active compounds are formed. The degradation process of the S-alk(en)ylcysteine sulfoxides is exemplarily shown for alliin in Figure 8. Upon ingestion a mixture of sulfur-containing compounds is taken in. To the best of our knowledge, there have been no studies regarding the metabolism of ramson in the human body. However, consumption of ramson and garlic leads to excretion of the same volatile compounds, both via urine and milk. It is conceivable that the metabolism of ramson is related to the metabolism of garlic and follows the same routes.

As discussed in our previous publication the sulfurous compounds containing a dithioallyl-group, e.g. DADS, are transformed to allyl mercaptan which is subsequently methylated to AMS (Germain et al., 2002; Lawson and Wang, 2005). The formation of AMS after garlic consumption was previously confirmed by our group (Scheffler et al., 2016a,b). Now, we can also confirm the presence of AMS in human milk and urine after ramson consumption. Likewise, we demonstrated that AMSO and AMSO_2_ are metabolites of garlic as well as of ramson constituents. Importantly, the pronounced differences in the amounts of S-alk(en)yl sulfoxides in the garlic and ramson plant were reflected in the detected quantities of AMS, AMSO, and AMSO_2._ Moreover, we observed differences between test persons that consumed aliquots of the same ramson sample. Accordingly, these inter-individual differences are most likely related to the individual metabolic conversion steps and respective enzyme activities due to genetic polymorphisms of enzymes, as has already been reported for drugs (Evans and Relling, 1999). Furthermore, the physiological status of each individual such as body mass index may influence the metabolism of ramson components (Bachour et al., 2012). In case of the milk samples, lactation period might be another influencing factor since the milk composition changes continuously throughout lactation and is adapted to the age of the infant.

## Conclusion

The impact of ramson consumption on human milk and urine composition was investigated. Samples were collected after ramson consumption and were investigated in comparison to control samples obtained prior to ramson consumption by means of GC-MS/O and GC-GC-MS/O. The analyses revealed three ramson-derived metabolites, namely AMS, AMSO, and AMSO_2_. Furthermore, the excretion profiles of these metabolites in human milk and urine were investigated. Therefore samples were collected over a time interval of about 24 h in case of urine samples and up to 9 h in case of milk samples. In the milk sets we observed the maximum concentrations of the metabolites within 3 h after ramson consumption. Likewise, the highest AMS and AMSO concentrations in urine sets were observed within 2 h after ramson consumption. The maximum concentration of AMSO_2_ was reached about 2 to 4 h after ramson ingestion. Overall, the present study revealed that ramson constituents are excreted correspondingly to garlic constituents albeit at lower concentration levels, correspondingly to lower content of the respective precursor substances in ramson.

## Data availability statement

All datasets generated for this study are included in the manuscript and the supplementary files.

## Author contributions

LS, CS, and AB conceived and designed the experiments. LS performed the experiments. TA contributed with analyses of ramson samples. LS analyzed the data. AB contributed reagents materials analysis tools. LS and CS conceived the publication that was approved by AB. All authors have read and approved the final manuscript.

### Conflict of interest statement

The authors declare that the research was conducted in the absence of any commercial or financial relationships that could be construed as a potential conflict of interest.

## Abbreviations

AMDS: allyl methyl disulfide
AMS: allyl methyl sulfide
AMSO: allyl methyl sulfoxide
AMSO_2_: allyl methyl sulfone
APDS: allyl propyl disulfide
APS: allyl propyl sulfide
DADS: diallyl disulfide
DAS: diallyl sulfide
DASO: diallyl sulfoxide
DASO_2_: diallyl sulfone
DATS: diallyl trisulfide
DCM: dichloromethane
DMDS: dimethyl disulfide
DMTS: dimethyl trisulfide
DPDS: dipropyl disulfide
DPTS: dipropyl trisulfide
FID: flame ionization detector
HRGC-GC-MS/O: two-dimensional high-resolution gas chromatography-mass spectrometry/olfactometry
HRGC-MS: high-resolution gas chromatography-mass spectrometry
HRGC-O: high-resolution gas chromatography-olfactometry
MPDS: methyl propyl disulfide
MPTS: methyl propyl trisulfide
RI: (linear) retention index
SAFE: solvent assisted flavor evaporation
SIDA: stable isotope dilution assay
SIM: selected ion monitoring.
