# Supplementary material for: Identification and Quantification of Volatile Ramson-Derived Metabolites in Humans
Source: Front Chem. 2018 Sep 11;6:410. doi: 10.3389/fchem.2018.00410 (PMC6141758; doi:10.3389/fchem.2018.00410)
Supplement: Supplementary file 1 [file Table_1.DOCX]

Supplementary Material

Identification and Quantification of Volatile Ramson-derived Metabolites in Humans

Laura Scheffler, Andrea Buettner*

*** Correspondence:** Professor Dr. Andrea Buettner: andrea.buettner@fau.de

# Material and Methods

Material and methods are adapted from previous studies performed by our group (Scheffler *et al.*, 2016a; Scheffler *et al.*, 2016b) (Scheffler *et al*. 2018).

## Chemicals

Ammonium chloride (NH_4_Cl), dichloromethane (DCM), sodium chloride (NaCl) and anhydrous sodium sulfate (Na_2_SO_4_) were purchased from VWR (Darmstadt, Germany). DCM was freshly distilled prior to use. Creatinine, disodium hydrogen phosphate (Na_2_HPO_4_), sodium sulfite (NaSO_3_) and urea were purchased from Aldrich (Steinheim, Germany). The following reference compounds were obtained from Sigma-Aldrich (Steinheim, Germany): allyl methyl sulfide (AMS), allyl methyl sulfone (AMSO_2_), diallyl disulfide (DADS), diallyl sulfide (DAS), dimethyl disulfide (DMDS), dimethyl trisulfide (DMTS), dipropyl trisulfide (DPTS), methyl propyl disulfide (MPDS) and methyl propyl trisulfide (MPTS). The reference compounds allyl methyl disulfide (AMDS), allyl propyl disulfide (APDS), allyl propyl sulfide (APS) and dipropyl disulfide (DPDS) were supplied by abcr (Karlsruhe, Germany). The reference compound allyl methyl sulfoxide (AMSO) and the stable isotopically labeled standards ^2^H_3_-AMS, ^2^H_3_-AMSO and ^2^H_3_-AMSO_2_ were purchased from aromalab (Freising, Germany). The remaining reference compounds 2-vinyl-4H-1,3-dithiin, 3-vinyl-4H-1,2-dithiin, diallyl sulfone (DASO_2_), diallyl sulfoxide (DASO) and diallyl trisulfide (DATS) were synthesized as described in Scheffler *et al.* (2016b).

Ramson was purchased from local supermarkets (ebl-Naturkost, Erlangen, Aldi-Sued, Erlangen or Früchte Hübschmann, Fürth, Germany). The creatinine kit was obtained from Labor+Technik Eberhard Lehmann GmbH (Berlin, Germany), and the combi®screen test strips were from Analyticon Biotechnologies AG (Lichtenfels, Germany).

## Human milk samples

Human milk samples were obtained from 13 different mothers (age range 27-39 years, mean 33). The volunteers had no known illnesses and their milk production exceeded their infants’ need. The sampling took place 9 to 37 seven weeks postpartum (mean 19 weeks). Milk samples were collected using a mechanical breast milk pump (Medela^TM^ Harmony, Medela AG, Baar, Switzerland). To avoid sulfurous compounds in the milk samples that were not associated with ramson consumption, the test persons were asked to avoid food containing high amounts of sulfur substances (e.g. garlic, onion, ramson, chives, cabbage and leek) on the testing day and two preceding days. Additionally, they were asked to keep record of their food during these three testing days. At the testing day ramson was washed with tap water and ground with a kitchen chopper (mini chopper CH180, Kenwood, Neu-Isenburg, Germany). Each mother provided one milk sample before ramson consumption and three further samples after ramson ingestion. The samples were collected according to the normal lactation period of each mother and immediately analyzed. Four consecutive milk samples formed a milk set in each case. In a pre-test one mother consumed about 3 g of ramson. All other test persons consumed about 10 g of ramson.

Four milk sets were collected for identification experiments. They were labeled ‘*M*’ with Arabic numerals, e.g. *M 1-1* to M *1-4*. In case of the quantification experiments nine milk sets were gathered and labeled ‘*M*’ with Roman numerals, e.g. *M I‑1* to *M I-4*.

## **Human urine** samples

Human urine samples were obtained from four volunteers (age 24-28 years, mean 26, two females, two males). One volunteer conducted the whole experimental series four times, the other three volunteers participated twice. Urine samples were collected in sterile brown glass bottles. At the testing day as well as two days preceding the testing day, the test persons were instructed to avoid sulfurous compounds and to record consumed foods and beverages as described for the milk sampling (cf. chapter 1.2). On the testing day ramson was freshly washed, ground and aliquoted into portions of 10 g. Each volunteer provided one urine sample before and seven samples after ingestion of 10 g of ramson at about the following times: 0.5 h, 1 h, 2 h, 4 h, 6 h, 8 h and 24 h after ramson consumption. Eight consecutive urine samples comprised one urine set. The samples were kept frozen at -80 °C until further analysis.

To rule out illnesses of the test persons, the first sample of each set was tested with a dipstick test (Combi-Screen Plus, Analyticon Biotechnologies AG, Lichtenfels, Germany) which allowed simultaneous testing of the following urine parameters: ascorbic acid, bilirubin, blood, glucose, ketones, leucocytes, nitrite, pH, protein, specific gravity/ density and urobilinogen.

For identification experiments one urine set was collected. The respective samples were labeled *U 1-1* to *U 1‑8*. For quantification analyses nine urine sets were analyzed. The urine samples of each set were labeled “*U*” with Roman numerals (e.g. *U II-1* to *U II-8*) and according to their sampling time:

- Pre: 3 to 7 min before ramson consumption
- 0.5 h post: 0.45 h to 0.55 h after ramson consumption
- 1 h post: 1.00 h to 1.05 h after ramson consumption
- 2 h post: 1.95 h to 2.10 h after ramson consumption
- 4 h post: 4.00 h to 4.10 h after ramson consumption
- 6 h post: 6.00 h to 6.10 h after ramson consumption
- 8 h post: 8.00 h to 8.15 h after ramson consumption
- 24 h post: 23.95 h to 24.20 h after ramson consumption

*U II* to *U V* were obtained at the same testing day with each test person consuming a ramson portion of about 10 g. Likewise, *U VI* to *U IX* were provided at the same testing days.

## Aroma profile analysis of human milk samples

Sensory evaluation was performed on all human milk samples. A trained panel (3 to 9 panelists, age range 23-36 years, mean 28 years) of the University Erlangen-Nürnberg (Erlangen, Germany) conducted the evaluation. The panel was trained for at least three months in recognizing about 140 selected odorants according to their odor qualities and in naming these in an in-house developed flavor language (Spitzer and Buettner, 2010). The milk samples, presented in covered brown glass bottles (capacity 100 mL), were evaluated orthonasally but not retronasally due to work safety considerations. Based on sensory pre-evaluations and corresponding to the chosen attributes in Scheffler *et al.* (2016b), the following attributes were selected for evaluation: hay-like, fishy, fatty, rancid, sweaty, metallic, grassy-green, sweet, egg white-like, buttery, lactic and garlic‑/ cabbage-like. Panelists were asked to rate these attributes on a scale from 0 to 3, whereas 0 is referred to in case of ‘no perception’ and 3 in case of ‘strong perception’.

## Determination of creatinine content in urine samples

Creatinine levels were determined in each urine sample using a creatinine kit (Labor+Technik Eberhard Lehmann GmbH, Berlin, Germany). The determination is based on the reaction of creatinine and picric acid in alkaline solution. Under these conditions creatinine and picric acid form a complex, that can be measured photometrically at a wave length of 492 nm.

## Solvent-assisted flavour evaporation (SAFE) of volatiles

The volatile fraction of the urine and milk samples was isolated by means of Solvent-assisted flavor evaporation (SAFE)-distillation (Engel *et al.*, 1999). DCM was added to the sample at a ratio of 1:2 (DCM/sample; v/v). The solution was stirred for 30 min at room temperature. Thereafter the mixture was applied to SAFE-distillation at 50 °C. After completion of the sample, 2 x 10 mL of DCM were applied to ensure complete transition of all volatile components. After thawing the aqueous phase was extracted three times with 25 mL DCM. All organic phases were combined, dried over anhydrous Na_2_SO_4_ and concentrated to 100 µL by means of Vigreux distillation and subsequent micro-distillation at 50 °C (Bemelmans, 1979).

In case of the quantification experiments, isotopically labeled standards were added to the sample prior to the work up described above.

## High-resolution gas chromatography-olfactometry (HRGC-O)

To identify odor active compounds that can be associated with ramson consumption high-resolution gas chromatography (HRGC-O) was performed. A Trace Ultra GC (Thermo Finnigan, Dreieich, Germany) with a DB-5 capillary (30 m x 0.32 mm, film thickness 0.25 µm, J&W Scientific, Fisons Instruments, Mainz-Kastel, Germany) and a DB-FFAP (30 m x 0.32 mm, film thickness 0.25 µm, J&W Scientific) was used. At the end of the capillaries the effluent was split by a Y-splitter between a sniffing port and a flame ionization detector (FID). Two deactivated, uncoated fused silica capillaries (i.D. 0.32 mm) were used to connect the Y-splitter with the FID or the sniffing port. The FID and the sniffing port were held at 250 °C and 270 °C, respectively. Carrier gas was helium at a flow rate of 2.0 mL/min. The extract (2 µL) was injected manually at 40 °C using the cold-on-column technique (Schomburg *et al.*, 1977). Thereby the sample was injected directly on a pre-column which was preceding the main-capillary. The pre-column was an uncoated, deactivated fused silica capillary (2-3 m, i.D. 0.32 mm) that was changed regularly to avoid accumulations of contaminants. The starting temperature (40 °C) was held for 7 min, then the oven temperature was raised to 240 °C (DB-FFAP) or 250 °C (DB-5) at a rate of 8 °C/min. This temperature was held for 5 min. The DB-5 capillary was further heated to 300 °C at a rate of 25 °C/min. The final temperature was held for 5 min.

## High-**resolution** gas chromatography-mass spectrometry (HRGC-MS)

Mass spectra of the eluting compounds were obtained with an Agilent MSD quadrupole system (GC 7890A and MSD 5975C, Agilent Technologies, Waldbronn, Germany). The system was equipped with a GERSTEL CIS 4 injection system and GERSTEL MPS 2 autosampler (GERSTEL, Duisburg, Germany). The software used to record the mass spectra and perform the data analysis was MSD ChemStation E.02.00.493 (Agilent Technologies). The identification experiments were performed with a DB-FFAP and a DB-5 capillary (30 m x 0.25 mm, film thickness 0.25 µm, Agilent J&W Scientific, Santa Clara, USA). For quantification experiments a DB-FFAP was used as analytical capillary. An uncoated, deactivated fused silica capillary was used as a pre-column (2 – 3 m, i.D. 0.53 mm). Another uncoated, deactivated fused silica capillary (0.3 – 0.6 m, i.D. 0.25 mm) was used to connect the analytical capillary with the MS. Helium was used as carrier gas at a flow rate of 1.0 mL/min. EI mass spectra were generated at 70 eV ionization energy in full scan mode (mass-to-charge ratio (*m/z*) range 30 – 350) as well as in Selected Ion Monitoring (SIM) mode (cf. table 1, main script). The extracts (2 µL) were applied by the autosampler using the on-column technique. The same temperature gradients were applied as described for the GC‑O analysis (cf. chapter 1.7).

## Two-dimensional high-resolution gas chromatography-mass spectrometry/ olfactometry (HRGC-GC-MS/O) (Heart-cut)

To identify trace constituents a two-dimensional gas chromatographic system was used. The system consisted of two Varian 450 GCs in combination with a Varian 220 MS ion trap mass spectrometer (Varian, Darmstadt, Germany). The system was equipped with a GERSTEL CIS 3 injection system and a GERTEL MPS 2 auto sampler (both GERSTEL, Duisburg, Germany). The first GC was equipped with a multi-column switching system (MCS 2, GERSTEL, Duisburg, Germany). A cryo-trap system (CTS 1, GERSTEL, Duisburg, Germany) was used to connect the first and the second GC. As analytical capillaries a DB-5 (30 m x 0.32 mm, film thickness 0.25 mm (Agilent J&W Scientific, Santa Clara, USA); first oven) and a DB-FFAP (30 m x 0.25 mm, film thickness 0.25 mm (Agilent J&W Scientific, Santa Clara, USA); second oven) were used. An uncoated, deactivated fused silica capillary was used as pre-column (2 – 3 m, i.D. 0.53 mm). Helium was used as carrier gas and the flow rate was set at 2.5 mL/min. In the first oven, the effluent was split between an olfactory detection port (ODP, GERSTEL, Duisburg, Germany) and a FID, as well as a cryo-trap during the cut interval. In the second oven, the effluent was transferred to the MS. All split capillaries were made of uncoated, deactivated fused silica material. The FID and the sniffing port were held at 250 °C and 260 °C, respectively. EI mass spectra were generated at 70 eV ionization energy in full scan mode. The *m/z* range for AMS was set at *m/z* 30 – 100, for all other compounds (DMDS, DAS, APS, AMDS, MPDS, DMTS, AMSO, AMSO_2_, DADS, DPDS, APDS, 2-vinyl-4H-1,3-dithiin, 3-vinyl-4H-1,2-dithiin, DASO, DASO_2_, MPTS, DATS and DPTS) it was set at *m/z* 30-350. The cut time intervals on the main column were determined by injection of the respective reference substances. The samples were applied to the GC at 40 °C using the cold-on-column technique. For identification of AMS 4 µL was applied, for the remaining compounds 2 µL was applied. The temperature programs for identification of AMS were as follows: The starting temperature of the first oven was 40 °C. This temperature was held for 7 min and then raised to 300 °C at a rate of 20 °C/min. The final temperature was held for 5 min. The second oven started at a temperature of 40 °C, which was held for 7 min. Afterwards the temperature was raised to 240 °C at a rate of 20 °C/min. The final temperature was kept for 5 min. For the remaining compounds the following temperature gradients were used: The first oven started at 40 °C, which was held for 2 min. Then the temperature was raised to 250 °C at a rate of 8 °C/min, which was held for 5 min. Afterwards the temperature was raised further to 300 °C at a rate of 25 °C/min. This final temperature was held for 5 min. The second oven also started at 40 °C which was kept for 2 min. Then it was raised to 240 °C at a rate of 10 °C/min. The final temperature was kept for 5 min. The transfer line between the first and the second oven was held at 250 °C and was cooled down to -100 °C for trapping of the respective substances. In pre-experiments fragmentation of compounds were observed during reheating of the transferline. To avoid this fragmentation the temperature of the transferline was set to 100 °C in case of DADS, DMTS, AMSO, AMSO_2_, AMDS, 2-vinyl-4H-1,3-dithiin, 3-vinyl-4H-1,2-dithiin, DASO, DASO_2_, DATS, MPTS and DPTS.

The quantification of AMS was performed on a two-dimensional GC-MS system consisting of two Agilent 7890 B GCs in combination with an Agilent 5977 B MS (Agilent, Waldbronn, Germany). The system was equipped with a GERSTEL CIS 4 injection system and a GERSTEL MPS 2 auto sampler (both GERSTEL, Duisburg, Germany). A multi-column switching system (µMCS, GERSTEL, Duisburg, Germany) was installed in the first GC and a cryogenic-trap system (CTS 1, GERSTEL, Duisburg, Germyn) was used to connect both GCs. The same analytical capillaries as described above were used for analysis. An uncoated, deactivated fused silica capillary functioned as pre-column (2 – 3 m x 0.53 mm) as described previously. Helium was used as carrier gas at a constant flow rate of 2.5 mL/min in the first GC and 1.0 mL/min in the second GC. The effluent was split between a FID and an ODP (ODP 3, GERSTEL), as well as a cryotrap during cut intervals in the first oven using the µMCS. In the second oven the effluent was split between an ODP and the MS using a Y-splitter. All split capillaries were made of uncoated, deactivated fused silica material. The FID and both ODPs were held at 250 °C and 270 °C, respectively. Mass spectra were recorded at 70 eV in full scan mode (m/z range 30-100) as well as in SIM-mode (cf. table 1, main script). The temperature programs were as follows: Starting temperatures were 40 °C for both GCs. In the first GC this temperature was held for 8 min, in the second oven for 7 min. Thereafter it was raised to 300 °C in the first oven and to 240 °C in the second oven at a rate of 20 °C/min. The final temperatures were held for 5 min.

## Identification of metabolites

Ramson-derived metabolites were identified by comparing their retention indices (RI), their respective odor as well as their mass spectra with those of reference standards. RI values were calculated according to Van den Dool and Kratz (1963). They were calculated for two analytical capillaries of different polarities (DB-FFAP and DB-5). The odor quality was described as perceived at the sniffing port via GC-O and the mass spectra were generated by either HRGC-MS or HRGC-GC-MS/O. Comparison of the mass spectra of the analyte with the reference standard was performed using the NIST Mass Spectral Search Program (Version 2.0 d, National Institute of Standards and Technology, Gaithersburg, MD, USA).

## **Quantification** by stable isotope dilution assay (SIDA) and calculation of metabolite profiles

In precedent trials the amount of AMS, AMSO and AMSO_2_ in milk and urine samples after ramson ingestion were evaluated. According to these precedent trials DCM-solutions of the respective isotopically labeled standards were added to samples collected before and after ramson ingestion. The mixture was worked up by SAFE distillation as described above (cf. chapter 1.6). AMSO and AMSO_2_ were quantified by means of GC-MS measurements in SIM mode. The *m/z*-ratios 104 + 107 and 120 + 123 were selected for analyses of AMSO and AMSO_2_ and their respective labeled analogues. The quantification of AMS was carried out by GC-GC-MS in SIM mode. The selected *m/z*-ratios for AMS and its labeled standard were 88 and 91. Next to milk and urine samples, solutions for calibration curves were analyzed. These solutions comprised defined mixtures of analyte and isotopic labeled standards (w/w; AMS/^2^H_3_-AMS: 1:10, 1:5, 1:3, 1:2, 1:1, 2:1; AMSO/ ^2^H_3_-AMSO and AMSO_2_/ ^2^H_3_-AMSO_2_: 1:10, 1:5, 1:2, 1:1, 2:1, 3:1, 5:1). The calibration curves were calculated as function between the intensity ratios of standard to labeled standard and the respective mass ratios (cf. table 1, main script). The solutions of the calibration curves were prepared in triplicate at three different days. For quantification the average of these calibration curves was used. With the resulting calibration function, the known amount of isotopic labeled standard added to the sample and the intensity ratio of analyte to isotopic labeled standard, the amount of analyte in the sample was calculated. In order to express the concentration of ramson-derived metabolites as µg/kg milk or urine or µg/mmol creatinine, the calculated amounts of AMS, AMSO and AMSO_2_ were divided by the amount of investigated sample (in kg) or normalized to creatinine content as described above (cf. chapter 1.10).

# Supplementary Figures and Tables

## Supplementary Figures


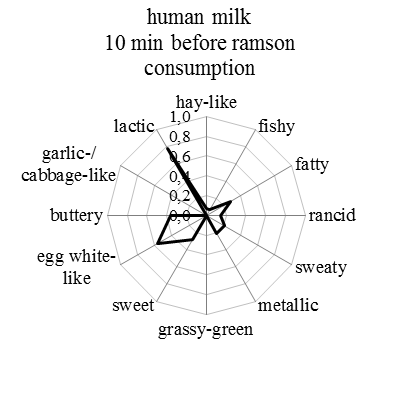

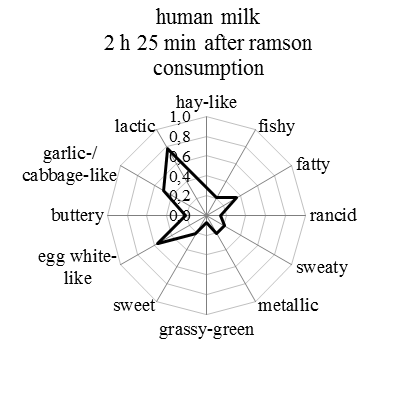

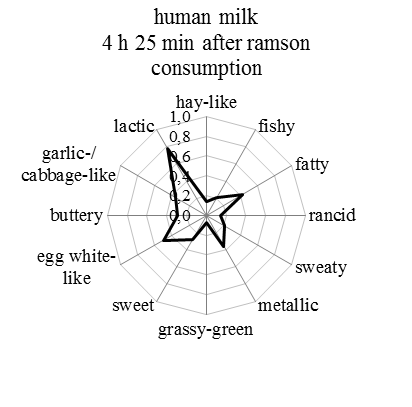

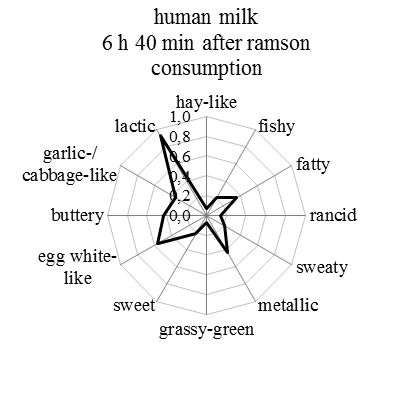


(B)


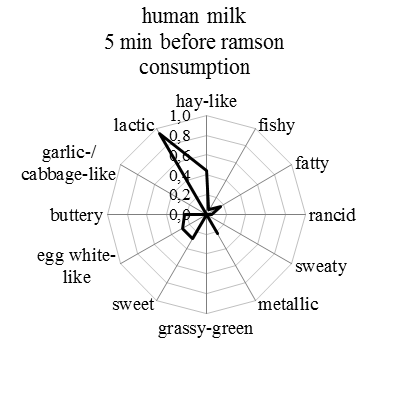

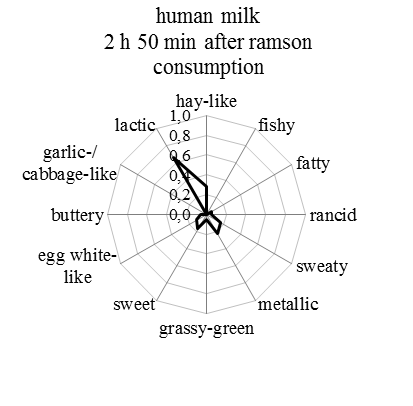

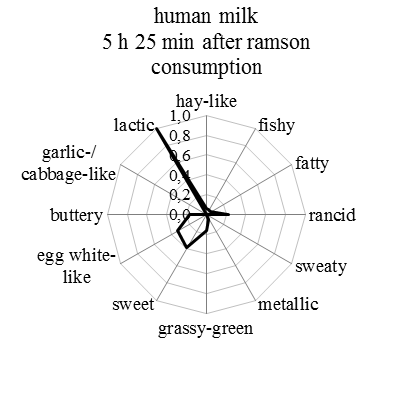

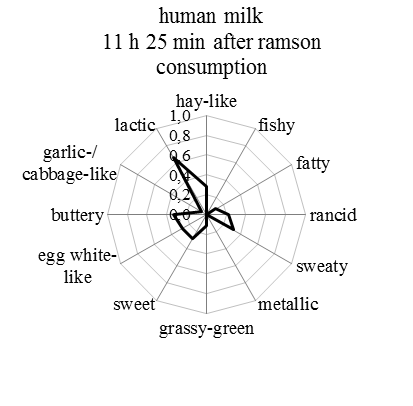


(A)

Figure S1: Odor profiles of human milk samples. (A) set *M 1*, (B) set *M 2*, (C) set *M 3* and (D) set *M 4*. The samples were collected at different times before and after ingestion of 3 g of ramson (A) or 10 g of ramson (B to D). Panelists were asked to rate the orthonasal perception on a scale from 0 (no perception) to 3 (strong perception). Values are mean ratings of all panelists. Note: The scale is only presented up to the value of 1 for better visualization.

Figure S1: continued


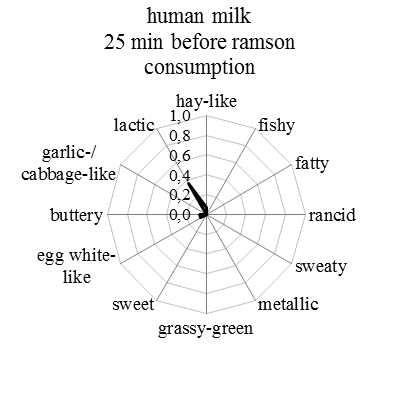

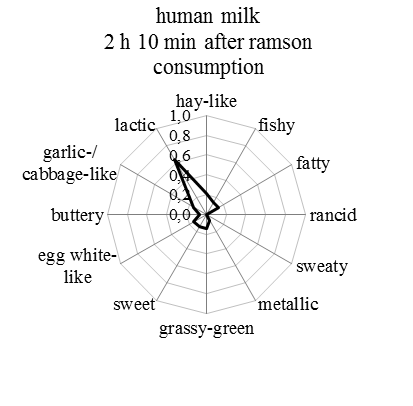

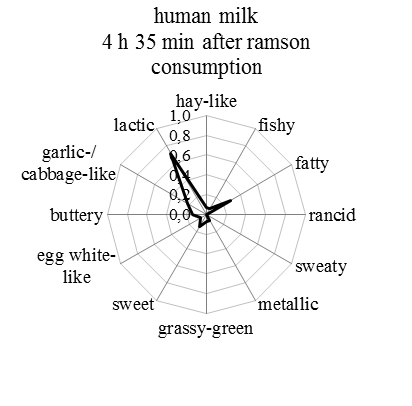

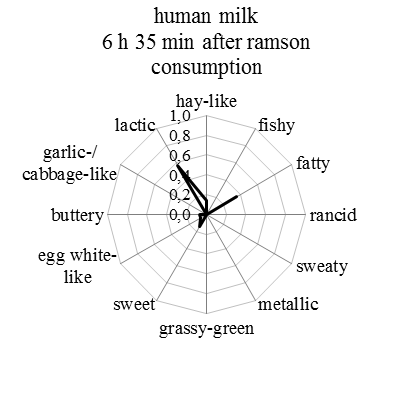


(C)


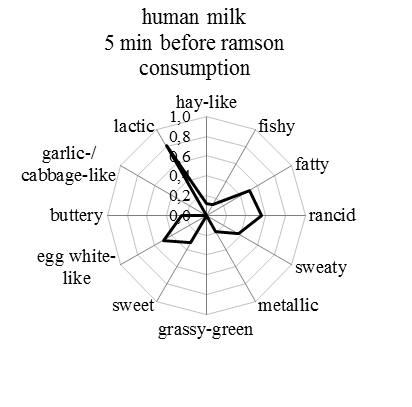

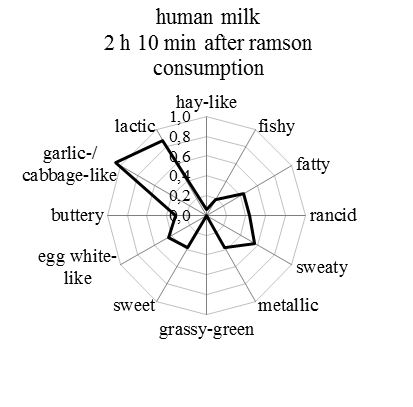

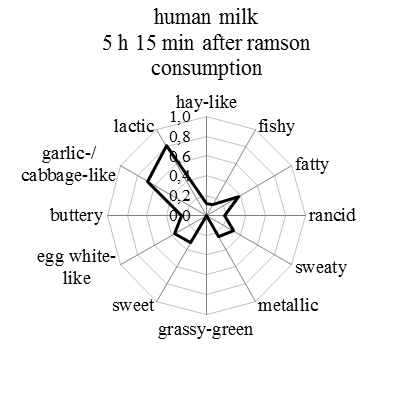

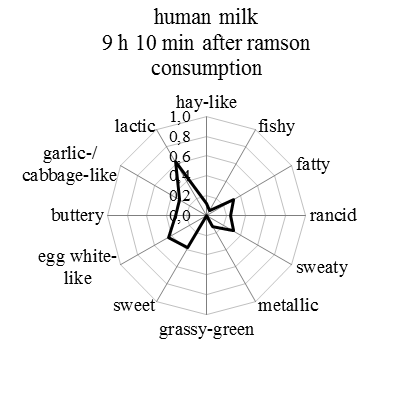


(D)

## Supplementary Tables

Table S1: Compilation of investigated human milk samples: time of milk sampling, amount of consumed ramson (g), mass (g) and volume (mL) of the investigated sample as well as AMS, AMSO and AMSO_2_ concentrations (µg/kg) of the milk samples.

| sample |  |  | sampling time |  | consumed ramson |  | quantity  milk sample | |  | AMS |  | AMSO |  | AMSO_2_ |
| --- | --- | --- | --- | --- | --- | --- | --- | --- | --- | --- | --- | --- | --- | --- |
|  |  |  | (h) |  | (g) |  | (g) | (mL) |  | (µg/kg) |  | (µg/kg) |  | (µg/kg) |
|  |  |  |  |  |  |  |  |  |  |  |  |  |  |  |
| M I | 1 |  | -0.08 |  | 10.3977 |  | 26.5439 | 26.0 |  | < LOD^1^ |  | < LOD^2^ |  | < LOD^3^ |
|  | 2 |  | 2.83 |  |  |  | 17.3655 | 17.0 |  | 1.92 |  | 58.05 |  | 98.60 |
|  | 3 |  | 6.08 |  |  |  | 13.3356 | 13.0 |  | 0.49 |  | 24.43 |  | 82.22 |
|  | 4 |  | 9.17 |  |  |  | 16.3803 | 16.0 |  | 0.57 |  | 10.01 |  | 48.23 |
| M II | 1 |  | -0.17 |  | 11.0455 |  | 40.0087 | 39.0 |  | < LOD^1^ |  | < LOD^2^ |  | < LOD^3^ |
|  | 2 |  | 2.00 |  |  |  | 30.7142 | 30.0 |  | 1.87 |  | 49.97 |  | 46.75 |
|  | 3 |  | 4.33 |  |  |  | 48.1106 | 47.0 |  | 0.98 |  | 18.45 |  | 51.39 |
|  | 4 |  | 6.67 |  |  |  | 51.2979 | 50.0 |  | 0.53 |  | 7.06 |  | 40.44 |
| M III | 1 |  | -0.83 |  | 9.9970 |  | 37.8464 | 37.0 |  | < LOD^1^ |  | < LOD^2^ |  | < LOD^3^ |
|  | 2 |  | 1.17 |  |  |  | 46.0661 | 45.0 |  | 0.34 |  | 32.69 |  | 26.61 |
|  | 3 |  | 3.50 |  |  |  | 35.8335 | 35.0 |  | 1.49 |  | 30.67 |  | 56.77 |
|  | 4 |  | 6.92 |  |  |  | 11.1993 | 11.0 |  | 0.34 |  | 8.79 |  | 35.96 |
| M IV | 1 |  | -0.08 |  | 10.4162 |  | 18.3899 | 18.0 |  | < LOD^1^ |  | < LOD^2^ |  | < LOD^3^ |
|  | 2 |  | 2.67 |  |  |  | 30.6854 | 30.0 |  | 0.40 |  | 36.18 |  | 31.95 |
|  | 3 |  | 5.25 |  |  |  | 18.4512 | 18.0 |  | 0.54 |  | 26.08 |  | 32.72 |
|  | 4 |  | 7.50 |  |  |  | 34.8946 | 34.0 |  | 0.41 |  | 16.45 |  | 27.90 |
| M V | 1 |  | -0.25 |  | 10.7080 |  | 31.6599 | 31.0 |  | 0.16 |  | 3.67 |  | traces^3^ |
|  | 2 |  | 1.50 |  |  |  | 42.7551 | 42.0 |  | 1.57 |  | 34.23 |  | 31.65 |
|  | 3 |  | 3.75 |  |  |  | 40.7718 | 40.0 |  | 0.88 |  | 17.88 |  | 30.93 |
|  | 4 |  | 5.92 |  |  |  | 51.0422 | 50.0 |  | 0.49 |  | 13.92 |  | 23.11 |

Table S1: continued

| M VI | 1 |  | -0.33 |  | 10.7292 |  | 34.8437 | 34.0 |  | 0.23 |  | 17.48 |  | 18.21 |
| --- | --- | --- | --- | --- | --- | --- | --- | --- | --- | --- | --- | --- | --- | --- |
|  | 2 |  | 1.50 |  |  |  | 34.6906 | 34.0 |  | 1.70 |  | 76.94 |  | 39.30 |
|  | 3 |  | 3.58 |  |  |  | 35.5624 | 35.0 |  | 2.01 |  | 59.54 |  | 50.49 |
|  | 4 |  | 5.50 |  |  |  | 14.6484 | 14.5 |  | 1.65 |  | 38.41 |  | 44.87 |
| M VII | 1 |  | -0.08 |  | 10.5049 |  | 51.3093 | 50.0 |  | < LOD^1^ |  | < LOD^2^ |  | < LOD^3^ |
|  | 2 |  | 1.92 |  |  |  | 32.6021 | 32.0 |  | 1.61 |  | 46.87 |  | 38.42 |
|  | 3 |  | 3.83 |  |  |  | 28.7067 | 28.0 |  | 0.92 |  | 42.37 |  | 54.68 |
|  | 4 |  | 7.17 |  |  |  | 21.5341 | 21.0 |  | 0.61 |  | 26.88 |  | 52.99 |
| M VIII | 1 |  | -0.08 |  | 10.5679 |  | 22.5652 | 22.0 |  | < LOD^1^ |  | < LOD^2^ |  | < LOD^3^ |
|  | 2 |  | 3.50 |  |  |  | 3.2144 | 3.2 |  | 0.52 |  | 33.79 |  | 41.51 |
|  | 3 |  | 5.92 |  |  |  | 34.8339 | 34.0 |  | 1.29 |  | 38.71 |  | 36.16 |
|  | 4 |  | 8.17 |  |  |  | 25.6555 | 25.0 |  | 0.37 |  | 9.35 |  | 27.77 |
| M IX | 1 |  | -0.08 |  | 10.7333 |  | 37.8622 | 37.0 |  | < LOD^1^ |  | < LOD^2^ |  | < LOD^3^ |
|  | 2 |  | 3.17 |  |  |  | 24.6069 | 24.0 |  | 0.73 |  | 75.36 |  | 50.91 |
|  | 3 |  | 5.75 |  |  |  | 19.3422 | 19.0 |  | 0.79 |  | 89.61 |  | 40.49 |
|  | 4 |  | 7.92 |  |  |  | 15.4075 | 15.0 |  | 0.31 |  | 28.72 |  | 28.07 |
| ^1^ LOD (AMS): 0.3 ng, LOQ (AMS): 1.1 ng ^2^ LOD (AMSO): 27.7 ng, LOQ (AMSO): 95.3 ng ^3^ LOD (AMSO_2_): 25.6 ng, LOQ (AMSO_2_): 89.4 ng  traces: > LOD and < LOQ  LOD and LOQ were determined in previous experiments (Scheffler et al. 2018) | | | | | | | | | | | | | | |

Table S2: Compilation of investigated urine samples: time of urine sampling, amount of consumed ramson (g), mass (g) and volume (mL) of the investigated sample as well as AMS, AMSO and AMSO_2_ (µg/kg and µg/mmol creatinine) of the urine samples.

| sample | |  | sampling time |  | consumed ramson |  | quantity  urine sample | |  | AMS | |  | AMSO | |  | AMSO_2_ | |
| --- | --- | --- | --- | --- | --- | --- | --- | --- | --- | --- | --- | --- | --- | --- | --- | --- | --- |
|  |  |  | (h) |  | (g) |  | (g) | (mL) |  | (µg/kg) | (µg/mmol creatinine) |  | (µg/kg) | (µg/mmol creatinine) |  | (µg/kg) | (µg/mmol creatinine) |
| U I^a^ | pre |  | -0.05 |  | 10.4223 |  | 50.0360 | 50.0 |  | < LOD^1^ | < LOD^1^ |  | < LOD^2^ | < LOD^2^ |  | < LOD^3^ | < LOD^3^ |
|  | 0.5 h post |  | 0.50 |  |  |  | 49.9726 | 50.0 |  | 0.30 | 0.35 |  | 41.42 | 48.58 |  | 16.47 | 19.33 |
|  | 1 h post |  | 1.00 |  |  |  | 49.9364 | 50.0 |  | 0.54 | 0.71 |  | 81.15 | 106.93 |  | 54.57 | 71.99 |
|  | 2 h post |  | 2.00 |  |  |  | 49.9225 | 50.0 |  | 0.46 | 0.63 |  | 62.47 | 85.93 |  | 70.95 | 97.74 |
|  | 4 h post |  | 4.00 |  |  |  | 50.0376 | 50.0 |  | 0.63 | 0.25 |  | 60.40 | 23.93 |  | 86.48 | 34.24 |
|  | 6 h post |  | 6.00 |  |  |  | 50.2993 | 50.0 |  | 0.36 | 0.07 |  | 34.69 | 7.08 |  | 84.63 | 17.18 |
|  | 8 h post |  | 8.00 |  |  |  | 50.1635 | 50.0 |  | 0.22 | 0.06 |  | 18.04 | 4.78 |  | 59.14 | 15.61 |
|  | 24 h post |  | 24.20 |  |  |  | 50.6058 | 50.0 |  | traces^1^ | traces^1^ |  | 5.98 | 0.58 |  | 6.44 | 0.62 |
| U II^b^ | pre |  | -0.08 |  | 10.8133 |  | 45.5486 | 45.0 |  | < LOD^1^ | < LOD^1^ |  | < LOD^2^ | < LOD^2^ |  | < LOD^3^ | < LOD^3^ |
|  | 0.5 h post |  | 0.50 |  |  |  | 26.9738 | 27.0 |  | traces^1^ | traces^1^ |  | 33.00 | 19.15 |  | 15.46 | 8.98 |
|  | 1 h post |  | 1.00 |  |  |  | 49.9397 | 50.0 |  | 0.35 | 0.28 |  | 46.30 | 36.92 |  | 35.16 | 28.07 |
|  | 2 h post |  | 2.00 |  |  |  | 49.9054 | 50.0 |  | 0.33 | 0.24 |  | 39.61 | 29.37 |  | 38.76 | 28.79 |
|  | 4 h post |  | 4.00 |  |  |  | 50.0770 | 50.0 |  | 0.34 | 0.10 |  | 30.54 | 8.50 |  | 41.74 | 11.60 |
|  | 6 h post |  | 6.00 |  |  |  | 50.1161 | 50.0 |  | 0.19 | 0.05 |  | 13.41 | 3.55 |  | 32.43 | 8.56 |
|  | 8 h post |  | 8.00 |  |  |  | 50.1455 | 50.0 |  | traces^1^ | traces^1^ |  | 10.34 | 3.25 |  | 23.74 | 7.44 |
|  | 24 h post |  | 24.00 |  |  |  | 40.3657 | 40.0 |  | < LOD^1^ | < LOD^1^ |  | 2.83 | 0.22 |  | traces^3^ | traces^3^ |
| U III^b^ | pre |  | -0.07 |  | 10.8281 |  | 50.0812 | 50.0 |  | < LOD^1^ | < LOD^1^ |  | < LOD^2^ | < LOD^2^ |  | < LOD^3^ | < LOD^3^ |
|  | 0.5 h post |  | 0.50 |  |  |  | 49.9504 | 50.0 |  | 0.22 | 0.22 |  | 28.57 | 28.49 |  | 6.94 | 6.93 |
|  | 1 h post |  | 1.00 |  |  |  | 49.9057 | 50.0 |  | 0.32 | 0.37 |  | 49.56 | 57.89 |  | 18.17 | 21.26 |
|  | 2 h post |  | 2.00 |  |  |  | 49.9102 | 50.0 |  | 0.36 | 0.43 |  | 56.85 | 68.78 |  | 35.52 | 43.05 |
|  | 4 h post |  | 4.00 |  |  |  | 50.0762 | 50.0 |  | 0.45 | 0.27 |  | 66.06 | 40.09 |  | 56.10 | 34.00 |
|  | 6 h post |  | 6.02 |  |  |  | 50.1634 | 50.0 |  | 0.87 | 0.30 |  | 52.77 | 17.97 |  | 65.81 | 22.33 |
|  | 8 h post |  | 8.00 |  |  |  | 50.3048 | 50.0 |  | 0.32 | 0.09 |  | 30.74 | 8.26 |  | 60.92 | 16.28 |
|  | 24 h post |  | 24.02 |  |  |  | 50.1084 | 50.0 |  | traces^1^ | traces^1^ |  | traces^2^ | traces^2^ |  | 5.20 | 0.95 |

Table S2: continued

| U IV^b^ | pre |  | -0.07 |  | 10.6621 |  | 50.0308 | 50.0 |  | < LOD^1^ | < LOD^1^ |  | < LOD^2^ | < LOD^2^ |  | < LOD^3^ | < LOD^3^ |
| --- | --- | --- | --- | --- | --- | --- | --- | --- | --- | --- | --- | --- | --- | --- | --- | --- | --- |
|  | 0.5 h post |  | 0.5 |  |  |  | 49.9763 | 50.0 |  | 0.46 | 0.30 |  | 50.68 | 32.29 |  | 18.51 | 11.80 |
|  | 1 h post |  | 1 |  |  |  | 49.9041 | 50.0 |  | 0.30 | 0.36 |  | 46.55 | 56.18 |  | 29.71 | 35.92 |
|  | 2 h post |  | 2.00 |  |  |  | 49.9366 | 50.0 |  | 0.27 | 0.34 |  | 36.56 | 45.73 |  | 38.56 | 48.29 |
|  | 4 h post |  | 4.00 |  |  |  | 49.9240 | 50.0 |  | 0.26 | 0.18 |  | 31.15 | 22.26 |  | 54.19 | 38.78 |
|  | 6 h post |  | 6.00 |  |  |  | 50.1200 | 50.0 |  | traces^1^ | traces^1^ |  | 21.83 | 6.91 |  | 52.23 | 16.50 |
|  | 8 h post |  | 8.00 |  |  |  | 50.1644 | 50.0 |  | traces^1^ | traces^1^ |  | 9.81 | 3.49 |  | 36.09 | 12.78 |
|  | 24 h post |  | 24.00 |  |  |  | 50.2259 | 50.0 |  | traces^1^ | traces^1^ |  | 0. traces^2^ | 0. traces^2^ |  | traces^3^ | traces^3^ |
| U V^b^ | pre |  | -0.10 |  | 10.6806 |  | 49.9976 | 50.0 |  | < LOD^1^ | < LOD^1^ |  | < LOD^2^ | < LOD^2^ |  | < LOD^3^ | < LOD^3^ |
|  | 0.5 h post |  | 0.53 |  |  |  | 49.9235 | 50.0 |  | 0.26 | 0.23 |  | 26.16 | 23.49 |  | 7.27 | 6.54 |
|  | 1 h post |  | 1.05 |  |  |  | 49.9292 | 50.0 |  | 0.40 | 0.37 |  | 46.51 | 42.84 |  | 19.69 | 18.17 |
|  | 2 h post |  | 2.03 |  |  |  | 49.9305 | 50.0 |  | 0.42 | 0.36 |  | 51.80 | 44.22 |  | 32.16 | 27.49 |
|  | 4 h post |  | 4.08 |  |  |  | 50.1545 | 50.0 |  | 0.52 | 0.14 |  | 51.51 | 13.52 |  | 49.67 | 13.00 |
|  | 6 h post |  | 6.08 |  |  |  | 49.9656 | 50.0 |  | 0.63 | 0.26 |  | 31.20 | 12.87 |  | 46.47 | 19.18 |
|  | 8 h post |  | 8.12 |  |  |  | 50.2047 | 50.0 |  | 0.27 | 0.07 |  | 23.81 | 6.12 |  | 49.34 | 12.63 |
|  | 24 h post |  | 24.07 |  |  |  | 50.1567 | 50.0 |  | traces^1^ | traces^1^ |  | 1.66 | 0.65 |  | 5.73 | 2.23 |
| U VI^c^ | pre |  | -0.08 |  | 10.6632 |  | 51.0996 | 50.0 |  | < LOD^1^ | < LOD^1^ |  | < LOD^2^ | < LOD^2^ |  | < LOD^3^ | < LOD^3^ |
|  | 0.5 h post |  | 0.50 |  |  |  | 50.1785 | 50.0 |  | 0.43 | 0.09 |  | 73.23 | 15.91 |  | 25.09 | 5.43 |
|  | 1 h post |  | 1.00 |  |  |  | 49.9836 | 50.0 |  | 0.40 | 0.26 |  | 51.71 | 32.88 |  | 35.36 | 22.49 |
|  | 2 h post |  | 2.00 |  |  |  | 49.8908 | 50.0 |  | 0.31 | 0.27 |  | 34.48 | 30.00 |  | 36.65 | 31.95 |
|  | 4 h post |  | 4.00 |  |  |  | 50.0670 | 50.0 |  | 0.32 | 0.10 |  | 27.01 | 8.60 |  | 39.02 | 12.41 |
|  | 6 h post |  | 6.00 |  |  |  | 50.4237 | 50.0 |  | 0.31 | 0.03 |  | 20.40 | 2.08 |  | 40.50 | 4.10 |
|  | 8 h post |  | 8.00 |  |  |  | 49.9718 | 50.0 |  | 0.41 | 0.30 |  | 8.87 | 6.45 |  | 26.11 | 18.98 |
|  | 24 h post |  | 23.92 |  |  |  | 50.6490 | 50.0 |  | traces^1^ | traces^1^ |  | traces^2^ | traces^2^ |  | 3.91 | 0.27 |

Table S2: continued

| U VII^c^ | pre |  | -0.08 |  | 10.6290 |  | 50.0145 | 50.0 |  | < LOD^1^ | < LOD^1^ |  | < LOD^2^ | < LOD^2^ |  | < LOD^3^ | < LOD^3^ |
| --- | --- | --- | --- | --- | --- | --- | --- | --- | --- | --- | --- | --- | --- | --- | --- | --- | --- |
|  | 0.5 h post |  | 0.50 |  |  |  | 49.9406 | 50.0 |  | 0.18 | 0.13 |  | 25.71 | 19.25 |  | 7.12 | 5.33 |
|  | 1 h post |  | 1.00 |  |  |  | 49.9427 | 50.0 |  | 0.30 | 0.28 |  | 48.93 | 46.35 |  | 19.87 | 18.84 |
|  | 2 h post |  | 2.00 |  |  |  | 49.9081 | 50.0 |  | 0.68 | 0.73 |  | 55.32 | 59.34 |  | 37.21 | 39.98 |
|  | 4 h post |  | 4.00 |  |  |  | 49.9493 | 50.0 |  | 0.31 | 0.31 |  | 39.48 | 39.74 |  | 45.50 | 45.84 |
|  | 6 h post |  | 6.00 |  |  |  | 49.9822 | 50.0 |  | 0.30 | 0.18 |  | 33.09 | 20.12 |  | 50.21 | 30.54 |
|  | 8 h post |  | 8.00 |  |  |  | 50.0227 | 50.0 |  | 0.23 | 0.13 |  | 27.13 | 15.35 |  | 50.66 | 28.65 |
|  | 24 h post |  | 24.02 |  |  |  | 49.9187 | 50.0 |  | traces^1^ | traces^1^ |  | 2.48 | 1.54 |  | 4.01 | 2.49 |
| U VIII^c^ | pre |  | -0.08 |  | 10.6735 |  | 50.0772 | 50.0 |  | < LOD^1^ | < LOD^1^ |  | < LOD^2^ | < LOD^2^ |  | < LOD^3^ | < LOD^3^ |
|  | 0.5 h post |  | 0.50 |  |  |  | 49.9236 | 50.0 |  | 0.25 | 0.30 |  | 38.62 | 46.81 |  | 12.94 | 15.71 |
|  | 1 h post |  | 1.00 |  |  |  | 49.8982 | 50.0 |  | 0.32 | 0.48 |  | 49.19 | 73.18 |  | 27.63 | 41.19 |
|  | 2 h post |  | 1.97 |  |  |  | 49.9145 | 50.0 |  | 0.33 | 0.57 |  | 44.63 | 76.69 |  | 40.15 | 69.11 |
|  | 4 h post |  | 4.00 |  |  |  | 50.0288 | 50.0 |  | 0.41 | 0.16 |  | 45.58 | 18.24 |  | 51.46 | 20.58 |
|  | 6 h post |  | 6.00 |  |  |  | 50.2880 | 50.0 |  | 0.61 | 0.12 |  | 34.50 | 6.62 |  | 68.09 | 12.98 |
|  | 8 h post |  | 8.00 |  |  |  | 50.1141 | 50.0 |  | 0.19 | 0.08 |  | 16.60 | 7.09 |  | 51.97 | 22.13 |
|  | 24 h post |  | 23.98 |  |  |  | 50.8070 | 50.0 |  | traces^1^ | traces^1^ |  | traces^2^ | traces^2^ |  | 2.38 | 0.19 |
| U IX^c^ | pre |  | -0.12 |  | 10.6857 |  | 50.1355 | 50.0 |  | traces^1^ | traces^1^ |  | 9.26 | 4.54 |  | 25.56 | 12.49 |
|  | 0.5 h post |  | 0.52 |  |  |  | 49.9648 | 50.0 |  | 0.28 | 0.34 |  | 27.93 | 34.10 |  | 26.97 | 32.96 |
|  | 1 h post |  | 1.00 |  |  |  | 49.9288 | 50.0 |  | 0.48 | 0.66 |  | 66.19 | 90.43 |  | 48.57 | 66.45 |
|  | 2 h post |  | 2.07 |  |  |  | 49.9638 | 50.0 |  | 0.64 | 0.61 |  | 54.79 | 52.03 |  | 55.00 | 52.28 |
|  | 4 h post |  | 4.07 |  |  |  | 50.2615 | 50.0 |  | 0.44 | 0.12 |  | 52.90 | 14.79 |  | 61.33 | 17.06 |
|  | 6 h post |  | 6.00 |  |  |  | 50.0714 | 50.0 |  | 0.28 | 0.10 |  | 26.98 | 9.34 |  | 53.20 | 18.39 |
|  | 8 h post |  | 8.10 |  |  |  | 50.0278 | 50.0 |  | 0.21 | 0.12 |  | 15.84 | 9.51 |  | 42.97 | 25.80 |
|  | 24 h post |  | 24.00 |  |  |  | 50.0243 | 50.0 |  | traces^1^ | traces^1^ |  | 7.28 | 4.71 |  | 22.46 | 14.50 |
| ^1^ LOD (AMS): 1.6 ng, LOQ (AMS): 7.6 ng ^2^ LOD (AMSO): 22.0 ng, LOQ (AMSO): 77.4 ng ^3^ LOD (AMSO_2_): 22.4 ng, LOQ (AMSO_2_): 78.6 ng  traces: > LOD and < LOQ  LOD and LOQ were determined in previous experiments (Scheffler et al. 2018) ^a^ … ^c^: same letters equal test persons that consumed ramson from the same ramson sample | | | | | | | | | | | | |  |  |  |  |  |

# Literature

Bemelmans, J.M.H. (1979). "Review of isolation and concentration techniques," in *Progress in Flavour Research, Proceedings of the 2nd Weurman Flavour Research Symposium held at the Univ. of East Anglia, Norwich, 2nd-6th April, 1978,* eds. D.G. Land & H.E. Nursten. (London: Applied Science Publ.), 79-98.

Engel, W., Bahr, W., and Schieberle, P. (1999). Solvent assisted flavour evaporation – a new and versatile technique for the careful and direct isolation of aroma compounds from complex food matrices. *European Food Research and Technology* 209(3-4)**,** 237-241. doi: 10.1007/s002170050486.

Scheffler, L., Sauermann, Y., Heinlein, A., Sharapa, C., and Buettner, A. (2016a). Detection of Volatile Metabolites Derived from Garlic (Allium sativum) in Human Urine. *Metabolites* 6(4). doi: 10.3390/metabo6040043.

Scheffler, L., Sauermann, Y., Zeh, G., Hauf, K., Heinlein, A., Sharapa, C., et al. (2016b). Detection of Volatile Metabolites of Garlic in Human Breast Milk. *Metabolites* 6(2)**,** 18.

Schomburg, G., Behlau, H., Dielmann, R., Weeke, F., and Husmann, H. (1977). Sampling techniques in capillary gas chromatography. *Journal of Chromatography A* 142**,** 87-102. doi: http://dx.doi.org/10.1016/S0021-9673(01)92028-X.

Spitzer, J., and Buettner, A. (2010). Characterization of aroma changes in human milk during storage at −19°C. *Food Chemistry* 120(1)**,** 240-246. doi: 10.1016/j.foodchem.2009.10.015.

van Den Dool, H., and Kratz, P.D. (1963). A generalization of the retention index system including linear temperature programmed gas—liquid partition chromatography. *Journal of Chromatography A* 11**,** 463-471. doi: http://dx.doi.org/10.1016/S0021-9673(01)80947-X.
